# Exploiting endogenous and therapy-induced apoptotic vulnerabilities in immunoglobulin light chain amyloidosis with BH3 mimetics

**DOI:** 10.1038/s41467-022-33461-z

**Published:** 2022-10-02

**Authors:** Cameron S. Fraser, Johan K. E. Spetz, Xingping Qin, Adam Presser, Jonathan Choiniere, Chendi Li, Stacey Yu, Frances Blevins, Aaron N. Hata, Jeffrey W. Miller, Gary A. Bradshaw, Marian Kalocsay, Vaishali Sanchorawala, Shayna Sarosiek, Kristopher A. Sarosiek

**Affiliations:** 1 John B. Little Center for Radiation Sciences, Harvard TH Chan School of Public Health, Boston, MA 02115 USA; 2 Program in Molecular and Integrative Physiological Sciences, Harvard TH Chan School of Public Health, Boston, MA 02115 USA; 3 Laboratory of Systems Pharmacology, Harvard Medical School, Boston, 02115 USA; 4 Massachusetts General Hospital Cancer Center, Charlestown, MA 02129 USA; 5 Department of Medicine, Harvard Medical School, Boston, MA 02115 USA; 6 Section of Hematology & Medical Oncology, Boston Medical Center, Boston, MA 02118 USA; 7 Amyloidosis Center, Boston University School of Medicine, Boston, MA 02118 USA; 8 Department of Biostatistics, Harvard TH Chan School of Public Health, Boston, MA 02115 USA; 9 Present Address: Department of Experimental Radiation Oncology, University of Texas MD Anderson Cancer Center, Houston, TX 77030 USA; 10 Present Address: Dana-Farber Cancer Institute, Harvard Cancer Center, Boston, 02215 USA

**Keywords:** Targeted therapies, Apoptosis, Predictive markers, Translational research

## Abstract

Immunoglobulin light chain (AL) amyloidosis is an incurable hematologic disorder typically characterized by the production of amyloidogenic light chains by clonal plasma cells. These light chains misfold and aggregate in healthy tissues as amyloid fibrils, leading to life-threatening multi-organ dysfunction. Here we show that the clonal plasma cells in AL amyloidosis are highly primed to undergo apoptosis and dependent on pro-survival proteins MCL-1 and BCL-2. Notably, this MCL-1 dependency is indirectly targeted by the proteasome inhibitor bortezomib, currently the standard of care for this disease and the related plasma cell disorder multiple myeloma, due to upregulation of pro-apoptotic Noxa and its inhibitory binding to MCL-1. BCL-2 inhibitors sensitize clonal plasma cells to multiple front-line therapies including bortezomib, dexamethasone and lenalidomide. Strikingly, in mice bearing AL amyloidosis cell line xenografts, single agent treatment with the BCL-2 inhibitor ABT-199 (venetoclax) produces deeper remissions than bortezomib and triples median survival. Mass spectrometry-based proteomic analysis reveals rewiring of signaling pathways regulating apoptosis, proliferation and mitochondrial metabolism between isogenic AL amyloidosis and multiple myeloma cells that divergently alter their sensitivity to therapies. These findings provide a roadmap for the use of BH3 mimetics to exploit endogenous and induced apoptotic vulnerabilities in AL amyloidosis.

## Abstract (web summary)

Immunoglobulin light chain amyloidosis is a lethal hematologic disorder driven by clonal plasma cells producing abnormal light chains that damage healthy tissues. Fraser *et al*. show that BH3 mimetics, which inhibit pro-survival proteins BCL-2 or MCL-1, can effectively eliminate diseased cells.

## Introduction

Immunoglobulin light chain (AL) amyloidosis is characterized by the production of monoclonal kappa or lambda-free light chains, which misfold and aggregate in tissues as insoluble amyloid fibrils, causing multi-organ dysfunction that can be rapidly fatal^1,2^. If diagnosed at late stages with advanced organ involvement, patients have a median overall survival (OS) of only 5–14 months^1^. Although half of the patients are diagnosed at earlier stages and can better tolerate treatment, this disease remains incurable, and median OS is poor^1^.

Therapy for AL amyloidosis is largely focused on the elimination of the underlying clonal plasma cells via risk-adapted treatment approaches based on the severity of organ involvement and the patient’s performance status. Low-risk patients (approximately 25% of those newly diagnosed) are often treated with high-dose melphalan followed by autologous stem cell transplantation (HDM/SCT)^3–5^. The remainder of patients with intermediate or high-risk disease or significant vital organ dysfunction are treated with a combination of therapies that may include proteasome inhibitors, alkylating agents, CD38 targeting antibodies and/or glucocorticosteroids^3,6–9^. Although multidrug regimens are preferred, often the highest risk patients can tolerate only low-dose or single agent therapy which is unlikely to control clonal plasma cells sufficiently and most patients, especially those with markers of resistance such as 1q21 or t(11;14), experience disease relapse resulting in significant morbidity or mortality^5,10–14^.

When effective, most anti-cancer therapies induce apoptotic cell death in neoplastic cells^15–19^ by modulating the expression and activity of the BCL-2 family of proteins that controls commitment to apoptosis. The mitochondrial apoptosis pathway is triggered when a pro-apoptotic, pore-forming protein (BAX or BAK) interacts with an “activator” BH3-only protein (BIM or BID) to cause mitochondrial outer membrane permeabilization (MOMP)^20^. MOMP results in the release of cytochrome c and other apoptogenic molecules from mitochondria, which activate caspases for cell elimination. However, pro-survival proteins in this family (e.g., BCL-2, BCL-X_L_, and MCL-1) can block apoptosis by inhibiting the activity of pro-apoptotic proteins via selective interactions. Finally, “sensitizer” pro-apoptotic proteins (e.g., BAD, Noxa, PUMA, HRK) are not efficient activators of BAX or BAK but can potently block pro-survival protein activity, which frees any bound pro-apoptotic proteins and pushes the cell toward apoptosis. In order for apoptosis to occur, the pro-survival proteins must be overwhelmed, and BAX/BAK activated.

Due to their prominent role in guiding cell fate in response to cell injury or stress, the state of the apoptosis pathway prior to treatment can strongly impact therapy responses and can be assessed using BH3 profiling^21^. The BH3 profiling assay measures the extent of mitochondrial outer membrane permeabilization (MOMP) and consequent cytochrome c release in response to pro-apoptotic BH3 peptides, which mimic the activity of full-length pro-apoptotic BH3-only proteins from the BCL-2 family^22,23^. The extent of MOMP induced by either promiscuous or highly selective pro-apoptotic BH3 peptides allows for the functional measurement of overall apoptotic priming (mitochondrial sensitivity to pro-apoptotic signaling) or dependence on specific pro-survival proteins, respectively. These dependencies can potentially be targeted by newly developed inhibitors. Notably, BH3 profiling has been used successfully to uncover apoptotic dependencies in several human cancers, including chronic lymphocytic leukemia^24,25^, acute myeloid leukemia^26,27^, lung cancers^28^, thyroid cancers^29^, and myeloproliferative neoplasms^30^.

The recent development of novel small-molecule inhibitors of the major pro-survival BCL-2 family proteins, called “BH3 mimetics”, has created an opportunity to therapeutically eliminate cell populations that are dependent on these proteins for survival. These agents have been transformative for the treatment of multiple hematologic malignancies^31–33^ and are currently being explored in other diseases^34–36^ and in combination with other therapies^37^. However, heterogeneity in apoptotic dependencies is common within and between cancer types. Clonal plasma cells in multiple myeloma have been found to be sensitive to inhibition of BCL-2 and MCL-1 at initial diagnosis, with increased MCL-1 dependence in relapsed disease, yet the heterogeneity in these dependencies has hampered treatment progress^38–40^. Although multiple myeloma and AL amyloidosis are related plasma cell disorders, the extent of apoptotic dependencies in AL amyloidosis has not been previously explored.

Here, we show that clonal plasma cells, derived from primary patient samples, are primed to undergo apoptosis and are sensitive to BH3 mimetics as single agents and in combination with current standard-of-care therapies. Sensitivity to the proteosome inhibitor bortezomib is enhanced by the BCL-2 inhibitor ABT-199 (Venetoclax) but not the MCL-1 inhibitor S63845 due to the bortezomib-induced increase in expression of the endogenous MCL-1 inhibitor Noxa. Additionally, we show that in vivo single-agent venetoclax treatment of mice implanted with AL amyloidosis cell line xenografts effectively reduces disease burden. Proteomic analysis indicates baseline and bortezomib treatment-induced differences in pathways related to metabolism, proliferation, and apoptosis between AL amyloidosis and multiple myeloma cell lines.

## Results

### Apoptotic sensitivity in AL amyloidosis

**Fig. 1:**
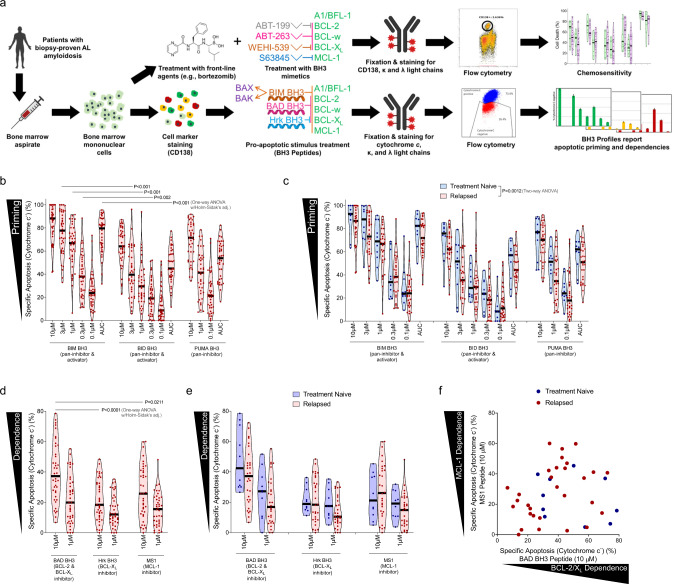
Plasma cells from patients with AL amyloidosis are primed for apoptosis and dependent on pro-survival proteins based on BH3 profiling. **a** Analysis overview: mononuclear cells were isolated from bone marrow aspirates from patients diagnosed with AL amyloidosis. One portion was stained with anti-CD38 and anti-CD138 antibodies then permeabilized with digitonin and exposed to BH3 peptides. After 60 minutes of exposure, cells were fixed and stained with anti-cytochrome c, anti-kappa and anti-lambda antibodies and analyzed by flow cytometry to determine the percentage of cells that had released cytochrome c (indicating MOMP) in response to each BH3 peptide. A second portion of cells were treated in vitro with drugs for either 24 or 48 h and then stained with anti-CD38, anti-CD138, anti-kappa, anti-lambda antibodies, as well as Annexin V. Viability of cells was then assessed by flow cytometry. Created with BioRender.com. **b** Mononuclear cells were isolated from patient-derived bone marrow aspirates across 40 patients that were not being treated at time of sample collection and BH3 profiled. Cells were exposed to pro-apoptotic BH3-only sensitizer peptides BIM, BID, and PUMA for 1 h, then cytochrome c loss was measured by flow cytometry in clonal plasma cells (*n* = 40). **c** Patient samples were stratified into treatment naïve or relapsed subgroups according to their treatment status at the time of collection (*n* = 9, *n* = 31). **d** Plasma cells were BH3 profiled and exposed to peptides that inhibit pro-survival proteins to determine anti-apoptotic protein dependence. **e** Patient samples were stratified based on their treatment status at the time of collection. **f** Comparison of BCL-2/BCL-X_L_ dependence versus MCL-1 dependence across 40 patients that were not being treated at time of sample collection. *P*-values were calculated using one-way ANOVA with Holm-Sidak’s adjustment for **b**, **d** and two-way ANOVA for **c**.

To elucidate the potential for using BH3 mimetics therapeutically in AL amyloidosis, we first sought to better understand how apoptosis is regulated in clonal plasma cells. We collected 88 bone marrow aspirates from patients with biopsy-proven AL amyloidosis – of these, eight specimens did not yield sufficient plasma cells for analysis, and twenty-two patients had no evidence of clonal plasma cell predominance in the bone marrow at time of sample collection and were thus excluded from further analysis (Fig. 1a). Of the remaining 58 samples that were used for these studies, 14 were from patients that were treatment-naïve and 44 were relapsed/refractory. Patients with the relapsed disease had been previously treated with standard agents, including proteasome inhibitors (bortezomib or ixazomib), glucocorticosteroids (dexamethasone), alkylating agents (cyclophosphamide or melphalan), anti-CD20 antibodies (rituximab), anti-CD38 antibodies (daratumumab), and/or immunomodulatory agents (pomalidomide or lenalidomide). Sixteen patients had prior HDM/SCT. At the time of analysis, all 58 samples were from patients with immunohistochemically detected lambda (λ) (47 samples) or kappa (κ) (11 samples) immunoglobulin light chain predominance in clonal plasma cells of the bone marrow. Additional markers of disease, such as elevated serum free light chains or abnormal serum or urine immunofixation electrophoreses were also present in most patients (see Supplementary Dataset 1 for patient details).

After collection, bone marrow aspirates were subjected to Ficoll-Paque separation to isolate mononuclear cells. A portion of mononuclear cells were taken for analysis by BH3 profiling to measure mitochondrial apoptotic priming and the extent of cellular dependence on pro-survival BCL-2 family proteins. Mononuclear cells were stained for CD138, permeabilized, and exposed to titrated doses of pro-death signaling peptides that target the intrinsic apoptosis pathway. After 60 minutes of exposure, the cells were fixed, and the extent of mitochondrial permeabilization induced by pro-apoptotic peptides was assessed by staining for cytochrome c and analysis via flow cytometry. We first analyzed only those patients that were not currently receiving treatment and found that clonal plasma cells from all samples released cytochrome c in response to pro-apoptotic BIM and BID BH3 peptides (10 µM) (Fig. 1b), which can inhibit all of the pro-survival BCL-2 family proteins^41^ and directly activate BAX or BAK^42^. While this confirms that AL plasma cells typically have an intact mitochondrial apoptosis pathway (express BAX or BAK at sufficient levels to undergo MOMP in response to a pro-apoptotic stimulus), the variability in responses suggests that differences in apoptotic sensitivity could affect clonal plasma cell responses to therapies. Further, the increased sensitivity of AL plasma cells to BIM compared to BID BH3 peptides, which is evident at most concentrations of either peptide or the area under the dose-response curve (AUC) summary metric, suggests they express higher levels of BAX than BAK due to the preferential interactions between BIM-BAX and BID-BAK^42^. Furthermore, most samples exhibited loss of cytochrome c in response to even mild doses of BIM or BID BH3 (1 µM or lower), indicating that these cells do not maintain a large reserve of unbound pro-survival proteins and are thus “primed for apoptosis”. This was confirmed by their high sensitivity to low doses of PUMA BH3 peptide (10 µM), which causes cytochrome c release by inhibiting pro-survival proteins and freeing any bound pro-apoptotic BH3-only activator proteins to then activate BAX or BAK^42^. The high sensitivity to PUMA BH3 peptide suggests that AL plasma cells would likely be dependent on pro-survival (anti-apoptotic) proteins and be susceptible to BH3 mimetics. Finally, we found that AL plasma cells from treatment-naïve patients were significantly more primed for apoptosis than those from patients that had relapsed disease (Fig. 1c), which would support increased resistance to apoptosis-inducing therapies in patients that have been previously treated. We did not detect significant differences in apoptotic priming in relapsed patients when comparing the treatments they received (Fig. S1a). Overall, the degree of priming detected in AL plasma cells is comparable to other hematological disorders^19,43,44^ and non-diseased hematopoietic cells^45,46^ and suggests that targeting apoptosis may be an effective therapeutic strategy for controlling AL amyloidosis.

### Plasma cell dependence on pro-survival BCL-2 family proteins

We next sought to assess the extent of plasma cell dependence on pro-survival proteins by measuring cytochrome c release in response to sensitizer BH3 peptides that inhibit the activity of specific pro-survival BCL-2 family proteins. For this analysis, we exposed permeabilized plasma cells to the BAD BH3 (inhibits BCL-2, BCL-W, and BCL-X_L_), HRK BH3 (inhibits BCL-X_L_) and MS1 (inhibits MCL-1) peptides. Although responses were heterogeneous between patient samples, we found that clonal plasma cells released cytochrome c in response to the BAD BH3 and MS1 peptides, indicating dependence on BCL-2/BCL-W/BCL-X_L_, or MCL-1, respectively (Fig. 1d). The responses to the HRK BH3 peptide were consistently lower than to the other peptides, suggesting that BCL-X_L_ has less anti-apoptotic activity in plasma cells and that the sensitivity to the BAD peptide is more an indication of BCL-2 dependence than BCL-X_L_ dependence. We also found that plasma cells from relapsed patients tended to be slightly less dependent on BCL-2 and more dependent on MCL-1 for survival, although the differences were not significant (Fig. 1e). We also noted a trend toward increased MCL-1 dependence in relapsed patients that had been treated with both HDM/SCT and a proteasome inhibitor as compared to either treatment alone (Fig. S1b). When comparing dependencies across samples (Fig. 1f), we found that although sensitivity to BAD and MS1 was similar in many patients, indicating dependence on both BCL-2/X_L_ and MCL-1, a subset of patient specimens exhibited increased sensitivity to one peptide over the other, indicating that dependencies can be preferential.

**Fig. 2:**
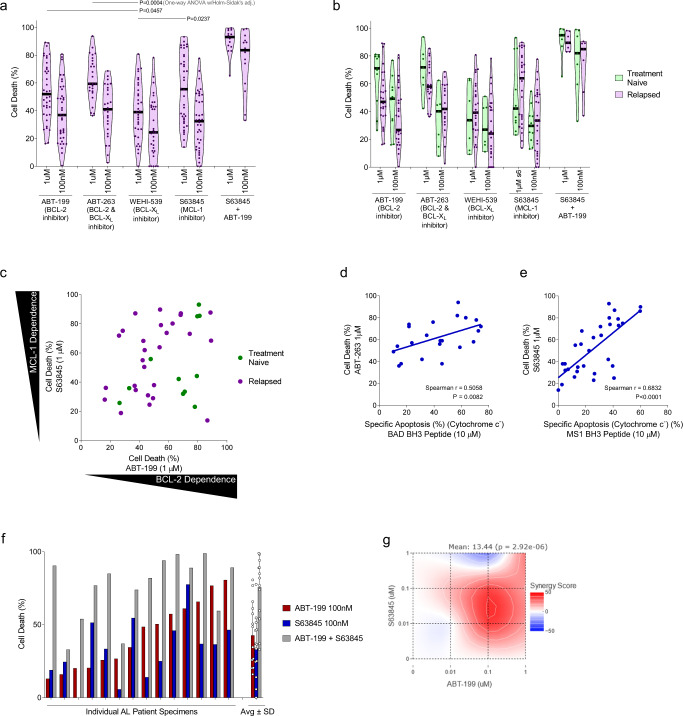
Plasma cells from patients diagnosed with AL amyloidosis undergo apoptosis in response to BH3 mimetics targeting BCL-2, MCL-1, and BCL-X_L_. BH3 profiling correlates with ex vivo sensitivity to specific BH3 mimetics. **a** Plasma cells were isolated from patient bone marrow aspirates, cultured ex vivo, and treated with BH3 mimetics. Cell death was measured by the loss of CD138^+^ cells after 24 h. **b** Patient samples were stratified into treatment naïve or relapsed subgroups according to their treatment status at the time of collection. Patients’ apoptotic response to BH3 mimetics indicated. **c** Comparison of BCL-2 dependence (apoptosis induced by ABT-199) versus MCL-1 dependence (apoptosis induced by S63845) across 31 patients that were not being treated at the time of sample collection. **d**–**e** The strength of correlation between clonal plasma cells’ apoptotic sensitivity via BH3 profiling at the time of isolation and their ex vivo chemosensitivity after 24 h ex vivo treatment were measured for **d** BAD BH3 peptide treatment (indicating BCL-2, BCL-X_L_ dependence) and ABT-263 treatment (BCL-2, BCL-X_L_ inhibitor), and **e** MS-1 peptide treatment (MCL-1 dependence) and 1 µM S63845 treatment (MCL-1 inhibitor). Spearman rho was used to test for correlation between responses to BH3 peptides and cellular sensitivity to indicated agents. **f** A subset of patient specimens were treated with both ABT-199 and S63845 and cell death was measured after 24 hours (*n* = 14). **g** HSA synergy analysis for combination ABT-199 and S63845 treatment. *P*-values were calculated using one-way ANOVA with Holm-Sidak’s adjustment for **a**.

Although BH3 profiling analysis allows for rapid measurement of apoptotic dependencies without in vitro culture, we also sought to test the sensitivity of clonal plasma cells to BH3 mimetics directly. We, therefore, treated BM-derived mononuclear cells with BH3 mimetics for 24 h in vitro and assessed viability by quantifying the loss of CD138-positive cells via flow cytometry (Fig. S1c). We found that AL plasma cells were highly sensitive to the BCL-2 inhibitor ABT-199 (venetoclax), the BCL-2/BCL-W/BCL-X_L_ inhibitor ABT-263 (navitoclax) and the MCL-1 inhibitor S63845 (Fig. 2a) but were less sensitive to the BCL-X_L_ inhibitor WEHI-539. These data were in agreement with our BH3 profiling data (Fig. 1c). In addition, we found that treatment-naïve patient plasma cells tended to be slightly more dependent on BCL-2 for survival while relapsed patient plasma cells were slightly more dependent on MCL-1 (Fig. 2b), demonstrating that single-agent MCL-1 inhibition may be effective even in the relapsed/refractory setting. We also noted that relapsed patients treated with both HDM/SCT and proteasome inhibitors were more MCL1 dependent than those treated with HDM/SCT alone (Fig. S1d), suggesting that different treatment regimens may drive clonal selection and apoptotic dependencies in divergent manners.

Direct sensitivity to BH3 mimetics (Fig. 2c) was similar to the results obtained via BH3 profiling, with plasma cells exhibiting the same heterogeneous patterns of dependencies on BCL-2 and MCL-1. In fact, we found that in vitro sensitivity to BH3 mimetics was correlated with sensitivity to sensitizer peptides in the BH3 profiling analysis when comparing BAD BH3 response to ABT-263 sensitivity (Fig. 2d) and when comparing MS1 peptide response to S63845 sensitivity (Fig. 2e).

We next sought to test the extent to which the heterogeneity in responses to individual BH3 mimetics could be overcome by co-treatment with both ABT-199 and S63845. This combination, even at low doses, was highly effective at inducing apoptosis in clonal plasma cells (Fig. 2g). Further, the activity of these agents was synergistic, as indicated by HSA synergy analysis (Fig. 2g). The synergy between these agents indicates that unbound MCL-1 actively sequesters the pro-apoptotic proteins being released by BCL-2 upon treatment with ABT-199 and vice versa. Thus, combination treatment prevents the cells from rapidly adapting to single-agent treatment by switching pro-apoptotic and pro-survival binding partners.

### BH3 mimetics in combination with standard therapies

**Fig. 3:**
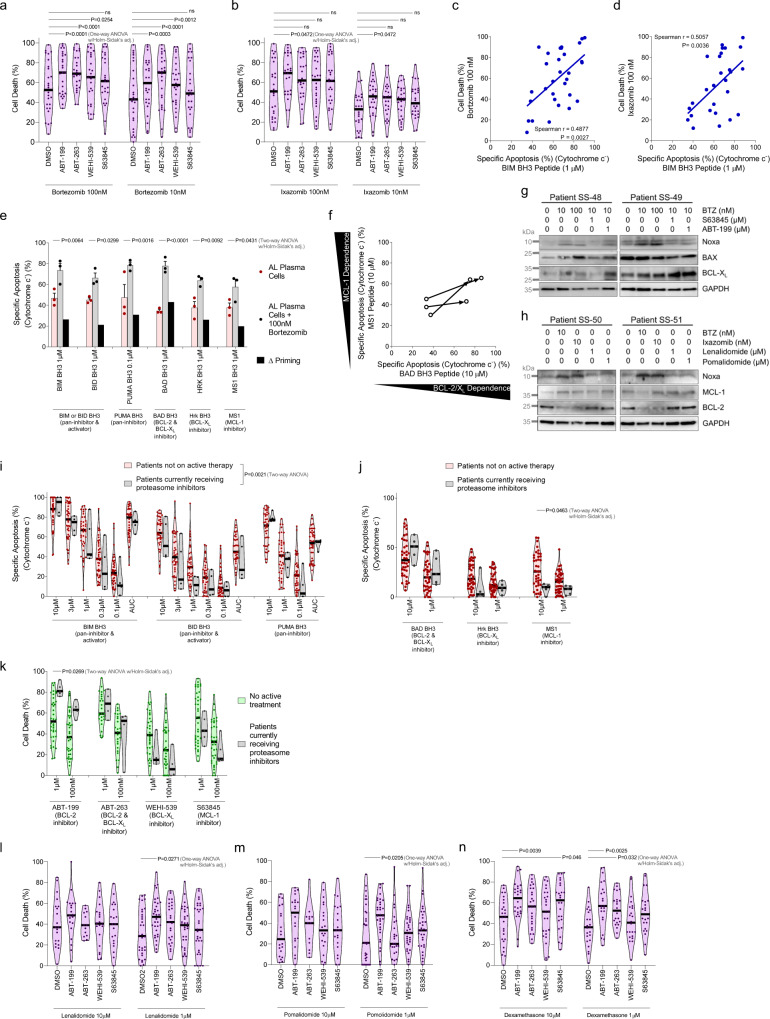
Plasma cells from patients with AL amyloidosis become more primed for apoptosis and more dependent on pro-survival BCL-2 family proteins when treated with bortezomib, which induces Noxa expression. **a**, **b** Plasma cells were isolated from patient bone marrow aspirates, cultured ex vivo, and treated with **a** bortezomib and **b** ixazomib as single agents or in combination with BH3 mimetics at 100 nM. Apoptosis was measured via Annexin V positivity after 24 h. **c**, **d** Ex vivo sensitivity of the clonal plasma cell population in response to **c** bortezomib and **d** ixazomib treatments measured via Annexin V positivity after 24 h correlated to apoptotic priming of clonal plasma cells at the time of isolation, measured by sensitivity to the BIM BH3 peptide via BH3 profiling. Spearman rho was used to test for correlation between responses to BH3 peptides and cellular sensitivity to indicated agents. **e**, **f** Plasma cells isolated from unique patients were cultured ex vivo with or without 100 nM bortezomib and BH3 profiled after 16 h. Data are presented as mean values + /- SEM (*n* = 3). **f** Treatment-induced changes in apoptotic dependencies. **g** Lysates were prepared from patient-derived bone marrow mononuclear cells and western blot analysis was performed looking at changes in expression of BCL-2 family proteins in response to 24 h of ex vivo culture with bortezomib treatment as a single agent or in combination with ABT-199 and S632845. **h** BCL-2 family protein expression was measured in response to 24 h ex vivo treatment with bortezomib, ixazomib, lenalidomide, or pomalidomide. **i**, **j** BH3 profiling and **k** chemosensitivity analysis of clonal plasma isolated from patients on active treatment with bortezomib. **l**–**n** Plasma cells were isolated from patient bone marrow aspirates, cultured ex vivo, and treated with **l** lenalidomide, **m** pomalidomide, or **n** dexamethasone as single agents or in combination with BH3 mimetics. *P*-values were calculated using one-way ANOVA with Holm-Sidak’s adjustment for **a**, **b**, **l**–**n**, two-way ANOVA with Holm–Sidak’s adjustment for **e**, **j**, **k**, and two-way ANOVA for **i**.

While the sensitivity of plasma cells to BH3 mimetics as single agents was encouraging, we also investigated the potential for combining these agents with standard-of-care therapies. Consistent with the strong activity of proteasome inhibitors as treatments for AL amyloidosis, we found that clonal plasma cells were sensitive to both bortezomib and ixazomib (Fig. 3a, b). Furthermore, sensitivity to proteasome inhibitors correlated with cellular sensitivity to the BIM BH3 peptide in the BH3 profiling assay (Fig. 3c, d), suggesting that cells that are more primed for apoptosis tend to be more sensitive to these agents, which induce apoptotic cell death by enhancing ER stress and inducing an unfolded protein response (UPR)^20,47,48^. This treatment is especially effective at inducing apoptosis in AL plasma cells due to the stress induced by the accumulation of light chains that are amyloidogenic^49^. Importantly, combining proteasome inhibition with ABT-199 and ABT-263 strongly enhanced cell death in clonal plasma cells (Fig. 3a, b) while adding S63845 to proteasome inhibition did not. This was unexpected due to the similar sensitivities to ABT-199 and S63845 when used as single agents (Fig. 2a). To determine the underlying mechanism that can impair additive induction of apoptosis through MCL-1 inhibition, we performed BH3 profiling on plasma cells undergoing bortezomib treatment. We found that bortezomib most strongly increased mitochondrial sensitivity to the BAD BH3 peptide and increased sensitivity to the HRK BH3 or MS1 peptides less so, indicating more of increased dependence on BCL-2 than BCL-X_L_ and MCL-1 (Fig. 3e, f). These results suggest that expression levels of BCL-2 family proteins may change during bortezomib treatment, as previously demonstrated in neoplastic cells^47,50^. Indeed, we found that proteasome inhibitors increased the expression of the pro-apoptotic BH3-only protein Noxa in all four patient samples that were evaluated via immunoblotting (Fig. 3g, h) while treatment with thalidomide derivatives or BH3 mimetics had no effect on the expression of this protein. The observed accumulation of Noxa, a protein with a short half-life^51^, is potentially due to inhibition of its constant degradation by the 26 S proteasome by bortezomib or via transcriptional upregulation in response to ER stress^52,53^. Increased expression of Noxa, an endogenous inhibitor of MCL-1, would effectively inhibit MCL-1 and prime cells for apoptosis, causing increased dependence on other pro-survival proteins that are expressed (in this case being BCL-2). In this manner, Bortezomib treatment can effectively mimic MCL-1 inhibition, potentially explaining the lack of increased apoptosis when combining MCL-1 inhibitors with proteasome inhibitors. To determine whether this would also be evident in patients, we BH3 profiled bone marrow aspirates from patients that were actively receiving bortezomib as a treatment for AL amyloidosis. Notably, although overall priming was lower in patients on bortezomib therapy (Fig. 3i), we found that BCL-2 dependence was significantly higher in these patients while MCL-1 dependence was decreased as measured by BH3 profiling (Fig. 3j). In agreement with BH3 profiling, clonal plasma cells from patients on bortezomib were more sensitive to ABT-199 ex vivo (Fig. 3k). These data are in agreement with our combination chemosensitivity data and suggest that inhibition of BCL-2, but not MCL-1, could enhance bortezomib-induced apoptosis.

Finally, we tested the in vitro sensitivity of the plasma cells to treatment with BH3 mimetics in combination with other standard therapies. BH3 mimetics increased clonal plasma cell sensitivity to low doses of the thalidomide derivatives lenalidomide and pomalidomide (Fig. 3l, m), as well as, dexamethasone (Fig. 3n), demonstrating that BH3 mimetics could potentially enhance clinical responses to these agents as well.

### Apoptotic dependencies in cell line models of AL amyloidosis and multiple myeloma

We next investigated the mechanisms driving these dependencies using cultured cell lines. We utilized the ALMC-1 and ALMC-2 cell lines, which were derived from a single patient that was initially diagnosed with AL amyloidosis, at which time the ALMC-1 cell line was established by culturing the clonal plasma cells in vitro with supportive cytokines^54^. After an initial response to treatment with dexamethasone and HDM/SCT, the patient relapsed with symptomatic multiple myeloma (MM), at which time the ALMC-2 cell line was established. Subsequent analysis of these clones showed that both cell lines are from a common ancestor, secrete λ free light chains that form fibrils, and were both likely present at the time of initial diagnosis^55^. Following patient treatment with dexamethasone, the ALMC-2 cells were likely selected based on their reported decreased sensitivity to this therapy and the expansion of this clone led to the manifestation of multiple myeloma. An additional consideration is that both cell lines secrete fibril-forming free light chains, which is not always observed in multiple myeloma. Therefore, these isogenic cell lines most accurately reflect λ free light chain secreting clonal plasma cells from newly diagnosed AL amyloidosis (ALMC-1) and multiple myeloma that was previously exposed to dexamethasone (ALMC-2).

**Fig. 4:**
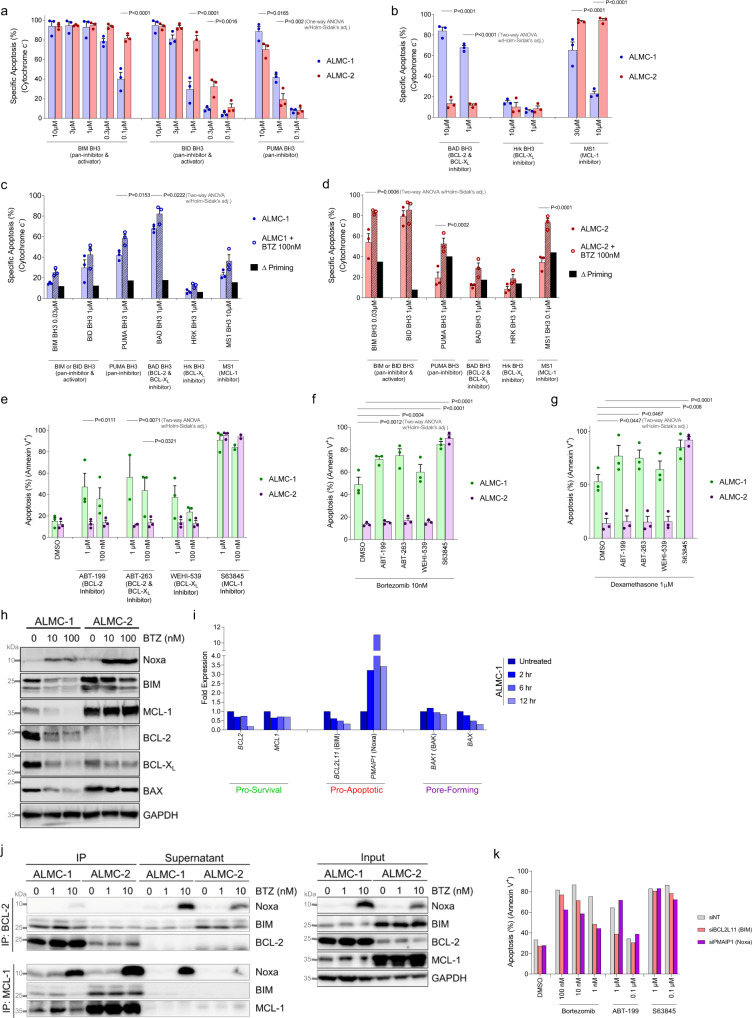
AL amyloidosis cell line ALMC-1 is sensitive to BCL-2 and MCL-1 inhibition while multiple myeloma cell line ALMC-2 is sensitive only to MCL-1 inhibition. **a**, **b** ALMC-1 and ALMC-2 cells were BH3 profiled with **a** activator or **b** sensitizer peptides at indicated concentrations (*n* = 3). **c**–**d** ALMC cell lines were BH3 profiled during short-term (4 h) treatment with bortezomib. Δ priming is calculated as the difference in treated versus untreated cells (*n* = 3). **e**–**g** ALMC cell lines were treated with indicated (**e**) single agents or **f–g** combinations for 12 h and apoptosis was detected by Annexin V staining (*n* = 3). **a**–**g** Data are presented as mean values + /- SEM. **h** Western blotting was performed on cell lines with and without 12 h treatment with bortezomib. **i** RT-qPCR validation of BCL-2 family gene expression in response to 10 nM bortezomib treatment, normalized to each gene’s untreated expression level (*n* = 3). **j** Immunoblot of BCL-2 and MCL-1 immunoprecipitation from ALMC-1 and ALMC-2 cells with and without 12 h bortezomib treatment. **k** In vitro sensitivity measured by Annexin V positivity of ALMC-1 cells treated for 12 h with indicated drugs, with BIM or Noxa knocked down by siRNA. *P*-values were calculated using one-way ANOVA with Holm-Sidak’s adjustment for **a** and two-way ANOVA with Holm–Sidak’s adjustment for **b**–**g**.

BH3 profiling of the ALMC-1 and ALMC-2 cell lines indicated that both cell lines were highly responsive to both pro-apoptotic activator peptides BIM and BID BH3 as well as the sensitizer peptide PUMA (Fig. 4a). Interestingly, ALMC-1 cells were less sensitive to the BIM and BID BH3 peptides while being more sensitive to PUMA BH3. This can be potentially due to differences in binding affinity in these peptides since BIM and BID are weaker BCL-2 inhibitors than PUMA^56^. This could alternatively be due to differences in expression of the pore-forming proteins since BIM and BID BH3 peptides can act as direct activators of BAX and BAK while PUMA BH3 peptide can only induce apoptosis by inhibiting pro-survival protein activity. Notably, while both cell lines exhibited dependence on MCL-1, the AL cell line also exhibited a dependence on BCL-2 while the MM cell line did not (Fig. 4b). Bortezomib treatment increased apoptotic priming in both cell lines and strengthened existing dependencies as measured by BH3 profiling (Fig. 4c, d). Consistent with these data, we found that ALMC-1 cells were sensitive to small molecule inhibitors of BCL-2 or MCL-1 while the ALMC-2 cells were sensitive only to MCL-1 inhibitors (Fig. 4e). Also, we found that BCL-2 inhibitors increased sensitivity to bortezomib (Fig. 4f) or dexamethasone (Fig. 4g) in ALMC-1 cells, which was consistent with our primary AL specimen data. Note that ALMC-1 cells are more sensitive than ALMC-2 cells to single agent treatment with bortezomib or dexamethasone, which is not unexpected given the ALMC-2 cells emergence after initial patient therapy.

Immunoblotting analysis of ALMC-1 and ALMC-2 cells demonstrated that bortezomib treatment again increased Noxa levels (Fig. 4h). Although impaired proteasomal degradation of Noxa could cause this short half-life protein to accumulate, we found that *PMAIP1*, which encodes Noxa, was strongly upregulated transcriptionally upon bortezomib treatment (Fig. 4i). This indicates that bortezomib-induced ER stress is triggering an unfolded protein response (UPR), which activates transcription of Noxa. We also noted that bortezomib treatment resulted in reduced levels of MCL-1, BCL-2 and BCL-X_L_ in ALMC-1 cells, some of which were also driven by transcriptional changes. However, bortezomib had no effect on MCL-1 protein levels in ALMC-2 cells – this may help explain why BCL-2 and BCL-X_L_ inhibition had little to no effect in the ALMC-2 cells, which likely express levels of MCL-1 sufficiently high to render inhibition of the other pro-survival proteins ineffective. Indeed, overall expression levels of MCL-1 were markedly higher in the MM cell line as compared to the AL cell line (Fig. 4h).

To further investigate the mechanisms driving differential responses to BH3 mimetics, we immunoprecipitated BCL-2 and MCL-1 to detect changes in binding partners upon bortezomib treatment. In ALMC-1 cells, we detected BIM bound to BCL-2 in both untreated cells and bortezomib-treated cells (Fig. 4j). However, while we detected BIM bound to MCL-1 in untreated cells, the Noxa that was upregulated in response to bortezomib treatment bound to MCL-1 and we no longer observed binding of BIM to MCL-1. These results indicate that bortezomib-induced Noxa is inhibiting the pro-survival activity of MCL-1 and freeing the normally bound BIM to activate apoptosis or bind to another free pro-survival protein (e.g., BCL-2) to shift the dependence. Notably, because Noxa is unable to activate BAX or BAK, subsequent treatment with an MCL-1 inhibitor would not be expected to strongly enhance apoptosis – this was also observed in our combination treatments of clonal plasma cells from primary specimens (Fig. 3a). The high expression levels of MCL-1 in ALMC-2 cells led to continued binding of BIM to MCL-1 despite Noxa upregulation – this explains the continued MCL-1 dependence of ALMC-2 cells even during bortezomib treatment.

We further confirmed the central roles of BIM and Noxa in bortezomib- and BH3 mimetic-induced apoptosis in ALMC-1 cells by knocking down their expression with siRNA (Fig. 4k). As expected, we found that knockdown of BIM reduced sensitivity to bortezomib, presumably by reducing the pool of potential pro-apoptotic activator proteins within the cells that are necessary to trigger BAX or BAK activation. This is consistent with BIM knockdown also causing a decrease in ABT-199 sensitivity. The high baseline expression of BIM in ALMC-1 cells is likely driven by ongoing ER stress, which is consistent with the high expression of the ER-stress associated master transcription factors ATF4 and CHOP in these cells as compared to lymphoma or multiple myeloma cells (Figure S1e) and previous reports linking ER stress to BIM upregulation^57–59^. Interestingly, BIM knockdown was not sufficient to impair S63845-induced apoptosis, suggesting that another pro-apoptotic binding partner such as BAX is bound to MCL-1 in untreated cells. Knockdown of Noxa also decreased bortezomib-induced apoptosis, confirming the important role of this protein in apoptosis resulting from ER stress. Due to its low expression in untreated cells (Fig. 4h), knockdown of Noxa did not alter sensitivity to single agent treatment with ABT-199 or S63845.

### ABT-199 (Venetoclax) induces AL amyloidosis remissions and extends survival in murine model

**Fig. 5:**
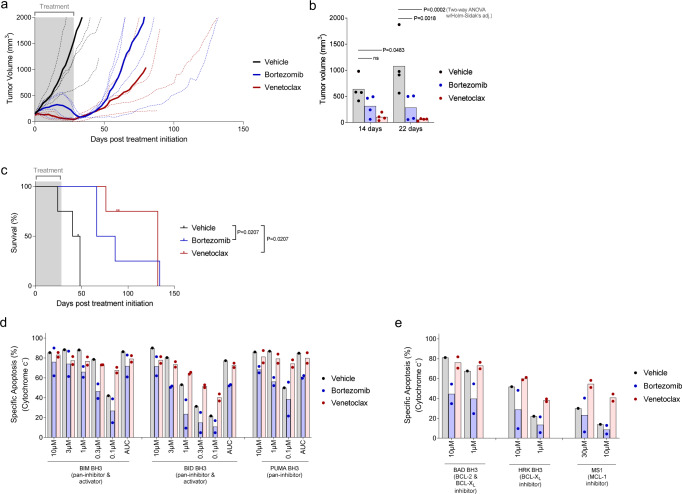
ABT-199 (Venetoclax) treatment in vivo reduces tumor burden and extends survival in NSG mice xenografted with ALMC-1 tumors. **a** Tumor growth curves of ALMC-1 tumors engrafted in NSG mice, treated with vehicle, venetoclax, or bortezomib. Thicker lines are average tumor size for each treatment (*n* = 4). **b** Tumor volume at the indicated number of days post initiation of treatment (*n* = 4). **c** Overall survival of mouse cohorts grouped by treatment received. **d**, **e** hCD45+ cells from ALMC-1 tumors from vehicle-treated (*n* = 1), bortezomib-treated (*n* = 2), and venetoclax-treated (*n* = 2) mice were BH3 profiled to measure their **d** overall level of apoptotic priming via sensitivity to the BIM BH3, BID BH3, and PUMA BH3 peptides and their **e** dependencies on the pro-survival BCL-2 family proteins BCL-2, BCL-X_L_, and MCL-1. *P*-values were calculated using two-way ANOVA with Holm–Sidak’s adjustment for **b** and Log-rank (Mantel-Cox) test to compare survival curves in **c**.

Although our results demonstrated that BCL-2 inhibitors effectively induce apoptosis in AL amyloidosis clonal plasma cells, we next investigated whether this would continue to be evident in vivo and how this therapy would compare with the standard of care, bortezomib. We, therefore, established flank xenografts of ALMC-1 cells and initiated treatment with either ABT-199 or bortezomib for a period of 28 days (Fig. 5a). Although there was some heterogeneity in tumor growth curves, bortezomib treatment halted the expansion of ALMC-1 xenografts by the third week of treatment in all four mice treated with this agent and doubled median survival (Fig. 5b). Although we expected to observe in vivo responses to ABT-199 treatment, we were surprised to see how effective this agent proved to be. ABT-199 treatment halted tumor growth almost immediately upon initiation of therapy, significantly reduced tumor growth compared to a vehicle faster than bortezomib (Fig. 5b) and tripled median survival compared with vehicle.

In a small subset of animals, we were able to remove xenografts and perform BH3 profiling of the cells that remained at the time of euthanasia. Interestingly, xenograft cells from animals treated with bortezomib were much less primed for apoptosis than those treated with vehicle (Fig. 5c), suggesting that this therapy either produced or, more likely, selected for apoptotic resistance, in agreement with our findings that samples from relapsed patients were less primed than treatment-naïve patient samples (Fig. 1c) Consistent with their overall lower priming, the cells were also less dependent on pro-survival proteins (Fig. 5d). In contrast, xenografts treated with ABT-199 tended to have increased priming compared to vehicle treatment. Further, at the time of euthanasia, the ABT-199 treated tumors maintained their BCL-2 dependence and even increased their dependence on MCL-1, though the limited sample size prevented robust statistical significance. The BH3 profiling data suggest that BCL-2 inhibitor treatment would continue to be effective if restarted and that treating tumors sequentially with ABT-199 and then S63845 may produce even stronger remissions.

### Mass spectrometry proteomics of AL and MM cell lines with bortezomib treatment

**Fig. 6:**
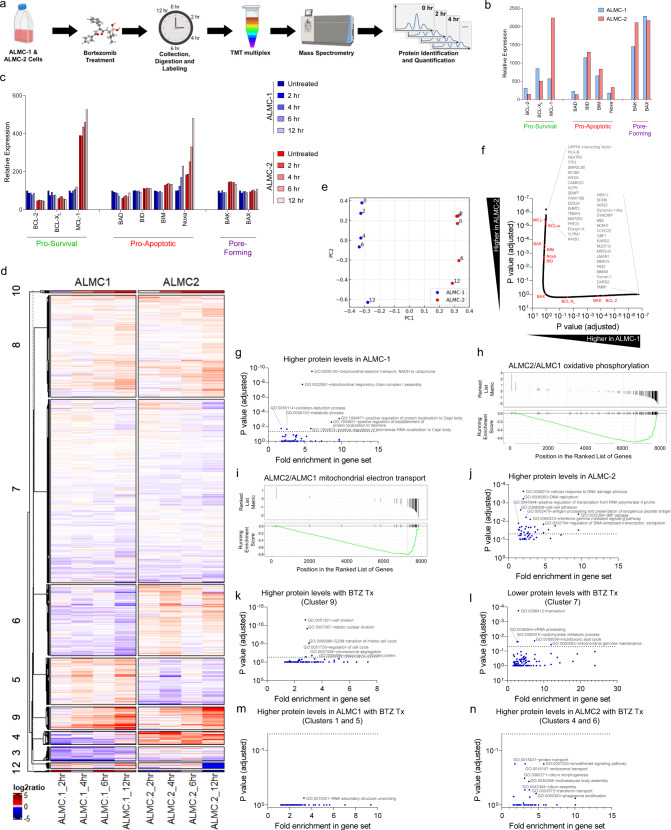
Proteomic analysis of bortezomib-treated ALMC-1 and ALMC-2 cells. **a** Overview of experiment and analysis to identify changes in protein expression associated with bortezomib treatment of AL cells. Created with BioRender.com. **b** Quantification of indicated BCL-2 family proteins. **c** Changes in BCL-2 family protein expression during bortezomib treatment. **d** Unbiased clustering analysis of proteins modulated by bortezomib treatment of ALMC-1 and ALMC-2 cell lines. Clustering was performed using the complete-linkage method based on Euclidean distance, and sub-clusters were separated by k means partitioning. **e** Principal component analysis of bortezomib-treated cells. **f** Ranking of all ~7900 quantified proteins with BCL-2 family proteins indicated in red. **g** Gene ontology (GO) biological processes (BP) that are associated with increased protein levels in ALMC-1 versus ALMC-2 cells. **h**–**i** Gene set enrichment analysis (GSEA) in ALMC-1 cells relative to ALMC-2 for proteins associated with **h** oxidative phosphorylation or **i** mitochondrial electron transport. **j** GO BP associated with increased protein levels in ALMC-2. **k**, **l** GO BP associated with **k** increasing or **l** decreasing protein levels during bortezomib treatment. **m**, **n** GO BP associated with increasing protein levels during bortezomib treatment specifically in ALMC-1 (**m**) or ALMC-2 (**n**) cells. *P-*values were calculated using linear regression and adjusted using Bonferroni correction for **f**, **g**, and **j**–**n**.

To further investigate the mechanisms driving these differences in sensitivity to bortezomib and BH3 mimetics in ALMC-1 and ALMC-2 cell lines, we used mass spectrometry to quantify differences in abundance of over 7900 proteins in untreated or bortezomib-treated cells (Fig. 6a). In untreated cells, we observed protein levels of BCL-2 family members that were in agreement with our immunoblotting results: BCL-2 and BCL-X_L_ levels were higher in ALMC-1 whereas MCL-1 expression was four-fold higher in the relapsed MM cell line (Fig. 6b). Abundance of pro-apoptotic and pore-forming proteins was largely similar between the two cell lines, yet we did note an increase in BAK expression in ALMC-2 cells – this could contribute to the higher sensitivity of these cells to BIM and BID peptides that we observed in BH3 profiling (Fig. 4a). These findings, along with our previous results, are consistent with reports describing MM as a predominantly MCL-1-dependent malignancy^38,60^ and highlights potential differences in dependencies on pro-survival proteins between these disorders. We next sought to determine how levels of BCL-2 family proteins were affected by proteasomal inhibition in these two cell lines. We treated the cells with bortezomib and collected cells at regular intervals over a 12 h treatment time course for quantitative mass spectrometry analysis. Notably, while we found that the level of Noxa was strongly increased by bortezomib in both cell lines, the relative amount of the pro-apoptotic sensitizer BH3-only protein BAD, which inhibits BCL-2, BCL-X_L_, and BCL-W but cannot activate BAX or BAK, was divergent: it decreased in ALMC-1 cells while increasing in ALMC-2 cells (Fig. 6c). The decrease in BAD levels in ALMC-1 cells would allow for increased sequestration of activator BH3-only proteins or the effectors BAX or BAK by BCL-2, BCL-X_L_, or BCL-W and could result in increased sensitivity to ABT-199, which is what we found in the chemosensitivity results for the AL but not MM cell lines. This is consistent with BCL-2 being a better therapeutic target in AL relative to MM, especially, in combination with proteasome inhibition.

To more broadly investigate commonalities and differences in the proteomes of ALMC-1 and -2 at baseline and during treatment with bortezomib (Fig. 6d), we carried out principal component analysis (PCA) across all perturbation conditions (Fig. 6e). The first principal component (PC1) robustly separated the two cell lines (ALMC-1 and ALMC-2), indicating that cell line-specific differences in protein levels were the largest overall separator in the proteomic datasets together with cellular transformation from AL to MM. The second principal component (PC2) seemed to capture the changes in protein expression during the treatment period (0, 2, 4, 6, and 12 h). Similar trends in PC2 and the similar start and end points of the time course indicated that bortezomib-induced changes could be similar and that induction of the ER stress response might be similarly regulated. However, we noted that ALMC-1 moved more quickly and further along the “time” axis, which may reflect increased sensitivity and accelerated commitment to apoptosis upon bortezomib treatment (Fig. 4f). These results indicate that both cell types respond similarly to proteasome inhibition and that the differences in protein levels at baseline (untreated) are more likely to determine whether apoptosis will be induced.

### GO pathway analysis shows differences in oxidative phosphorylation, MAPK/ERK signaling pathways

We next sought to identify the potentially informative changes in individual proteins as well as signaling pathways and biological processes that differ between the ALMC-1 and ALMC-2 cells at baseline and in response to bortezomib treatment. We first used a linear regression model on standardized mass spectrometry protein level data to test whether each protein has a significant positive (indicating increased levels in ALMC-1) or negative (indicating higher levels in ALMC-2) coefficient for each covariate (cell line, treatment duration, or both). We then ranked the genes based on their Benjamini–Hochberg (BH) corrected *p*-values (Fig. 6f). As expected based on our earlier analysis, the relative level of BCL-2 family proteins was significantly different between the two cell lines with MCL-1 exhibiting the largest difference (Fig. 6f, BCL-2 family proteins marked in red). We also found that several proteins that are integral to the translation of mitochondrially-encoded proteins were more abundant in ALMC-1 than ALMC-2 cells, including arginyl-tRNA synthetase 2, mitochondrial (RARS2), aspartyl-tRNA synthetase 2, mitochondrial (DARS2) and isoleucyl-tRNA synthetase 2, mitochondrial (IARS2), which suggests increased mitochondrial respiratory chain activity and oxidative phosphorylation in AL cells relative to MM. In contrast, proteins associated with mitogen-activated protein kinase (MAPK/ERK) signaling were more abundant in ALMC-2 cells, including mitogen-activated protein kinase kinase 3 (MAP2K3) and calcium/calmodulin-dependent protein kinase II gamma (CAMK2G). These data, along with enrichment analysis of MAPK and ERK signaling cascades (Figure S2a-b) suggest that aberrant MAPK/ERK signaling may drive uncontrolled cell proliferation in the transformed MM cells and is consistent with previous findings of MAPK activating mutations in this disease but not AL amyloidosis^61^.

To characterize signaling pathway differences between these cell lines at baseline, we performed gene ontology (GO) enrichment analysis from proteomic data. Consistent with our analysis above, we found that the ALMC-1 cells expressed increased levels of multiple proteins associated with mitochondrial electron transport and respiratory chain complex assembly as well as oxidation-reduction processes (Fig. 6g). Gene set enrichment analysis (GSEA) revealed extensive downregulation of nearly every protein associated with oxidative phosphorylation (Fig. 6h) and mitochondrial electron transport (Fig. 6i) in ALMC-2 cells relative to ALMC-1. These differences suggest that AL cells have higher rates of oxidative phosphorylation relative to MM cells, which is consistent with the increased reliance upon glycolysis and glutaminolysis in plasma cells that have transformed to MM^62^. We also found that ALMC-2 cells contain increased levels of proteins associated with DNA replication and gene transcription relative to ALMC-1, consistent with the higher proliferation rate of MM cells (Fig. 6j). Overall, these changes suggest that cellular transformation from AL to MM in these cells is associated with a glycolytic switch to support increased production of cellular components and cell cycling. Consistent with this, we found that ALMC-1 cells have increased rates of mitochondrial respiration relative to ALMC-2 cells (Fig. S2c, d).

We next identified biological processes that are affected by proteasome inhibition in AL and MM cells in a similar manner (indicated by cluster 9 in Fig. 6d for increasing expression, cluster 7 for decreasing). We detected higher levels of proteins associated with cell division, nuclear division, G2/M transition and chromosome segregation in bortezomib-treated cells – this is consistent with their accumulation due to treatment-induced cell cycle arrest (Fig. 6k). We also found that proteins associated with translation, rRNA processing and metabolism were reduced in bortezomib-treated cells, consistent with the activation of the unfolded protein response (Fig. 6l). Enrichment analysis for genes known to be associated with the unfolded protein response demonstrated that both cell lines express higher levels of UPR-associated proteins with similar dynamics, with ALMC-2 cells having a slightly accelerated response (Fig. S2e). We next identified proteins that are differently regulated during bortezomib treatment in the two cell lines. Interestingly, GO pathway analysis indicated that bortezomib treatment leads to higher expression of proteins associated with RNA processing and stability as well as NF-κB signaling in ALMC-1 but not ALMC-2 cells (clusters 1 and 5 in Fig. 6d) (Fig. 6m). In contrast, bortezomib treatment uniquely affects protein transport and endosome/phagosome function in ALMC-2 but not ALMC-1 cells and also increases levels of proteins associated with smoothened pathway signaling (clusters 4 and 6 in Fig. 6d) (Fig. 6n). Note that these changes were more moderate than the differences between the cell lines in the untreated state and did not reach statistical significance, again suggesting that baseline differences in protein expression between the two cell lines, such as differences in MCL-1 levels, are more likely to be major determinants of cell fate upon bortezomib treatment than differences in signaling as a consequence of proteasomal inhibition.

### Kappa/Lambda predominance, t(11:14) translocations and apoptotic dependencies

**Fig. 7:**
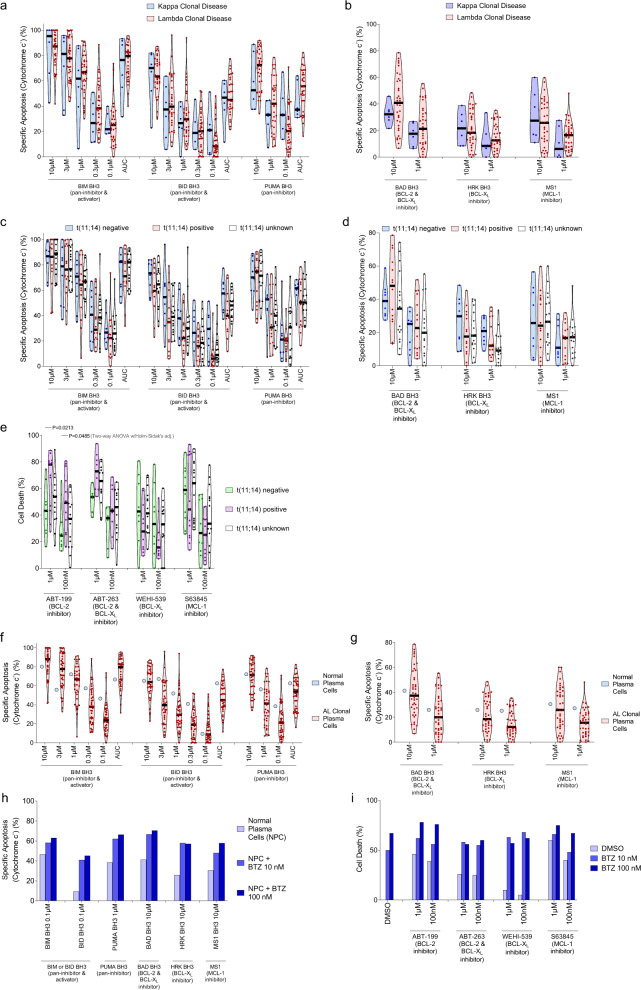
Markers of apoptotic vulnerabilities in AL amyloidosis. **a**, **b** Clonal plasma cells isolated from Kappa- and Lambda-predominant AL amyloidosis were isolated and BH3 profiled to measure their **a** overall level of apoptotic priming via sensitivity to the BIM BH3, BID BH3, and PUMA BH3 peptides and their **b** dependencies on the pro-survival BCL-2 family proteins BCL-2, BCL-X_L_, and MCL-1. **c**–**e** BH3 profiling indicating (**c**) overall apoptotic priming and **d** BCL-2 family dependencies in clonal plasma cells from AL amyloidosis patients with t(11;14), and **e** ex vivo sensitivity to BH3 mimetics. **f**, **g** BH3 profiling CD138 + cells from a healthy bone marrow donor showing (**f**) overall priming and **g** dependencies on pro-survival proteins compared to clonal AL CD138 + cells. **h** Dynamic BH3 profiling of normal plasma cells treated with bortezomib for 12 h. **i** Ex vivo sensitivity of normal plasma cells to the combination treatment of bortezomib with indicated BH3 mimetics. *P*-values were calculated using two-way ANOVA with Holm–Sidak’s adjustment for **e**.

AL amyloidosis is typically diagnosed as either a κ or λ predominant disease and clinical outcomes can differ depending on the disease isotype^63,64^. We found that apoptotic priming (Fig. 7a) and apoptotic dependencies (Fig. 7b) were not significantly different in κ and λ clonal plasma cells. We also compared apoptotic dependencies in subsets of specimens based on cytogenetic features, including translocation (11:14), which puts control of cell cycle-promoting cyclin D1 (*CCND1*) under the immunoglobulin heavy chain (*IGH*) promoter and has been previously associated with increased dependence on BCL-2 in MM^65^. Although we did not detect significant differences in overall priming (Fig. 7c) we did note a tendency for t(11;14) positive patients to have increased dependence on BCL-2 as measured by BH3 profiling (Fig. 7d) and significantly more sensitivity to ABT-199 and ABT-263 but not WEHI-539 or S63849 ex vivo (Fig. 7e). These results are in agreement with previous reports in multiple myeloma^65,66^ and suggest that t(11;14) translocations may be associated with increased dependence on BCL-2 and potentially used as a biomarker for assignment of therapy.

Finally, we also sought to compare apoptotic priming and dependencies between clonal plasma cells with plasma cells from healthy donors. Although we were only able to obtain one healthy bone marrow specimen, the priming (Fig. 7f) and dependencies (Fig. 7g) in normal plasma cells seem consistent with those detected in clonal plasma cells in AL. We also found that bortezomib treatment increased priming and dependencies on pro-survival proteins BCL-2 and MCL-1, suggesting that the results we observed in clonal plasma cells are likely due to a conserved signaling pathway in this cell lineage and exploited therapeutically for AL therapy (Fig. 7h). Indeed, the bortezomib-induced increase in priming and pro-survival protein dependency translated to increased sensitivity to BH3 mimetics in normal plasma cells when co-treated with bortezomib (Fig. 7i).

## Discussion

Our studies establish that clonal plasma cells have an intact and, in the majority of cases, primed apoptosis pathway. This indicates that therapeutic interventions that can enhance pro-apoptotic signaling are likely to be effective treatments in this disorder. We also found that plasma cells from treatment-naïve patients are more primed than those in relapsed disease, which is consistent with the previous reports^19,25,43^ and suggests that existing therapies preferentially eradicate cells that are most highly primed for apoptosis. This was directly evident in our in vivo studies, which demonstrated that xenografts growing out after bortezomib therapy exhibited a reduction in overall priming. We would therefore expect initial treatment with BH3 mimetics would lead to the strongest responses. Indeed, we found that single-agent treatment of AL amyloidosis xenografts with ABT-199 produced powerful regressions and tripled median survival. Finally, in the relapsed/refractory setting, we found that clonal plasma cells continued to exhibit dependence on BCL-2 and MCL-1, suggesting that BH3 mimetics and, especially, MCL-1 inhibitors would be effective in this patient population.

In results from in vitro chemosensitivity as well as BH3 profiling assays, which have both been previously shown to predict patient responses to BH3 mimetics^19,26,67,68^, we consistently found that clonal plasma cells in AL amyloidosis can be highly dependent on MCL-1, or BCL-2 for survival, or both. BCL-2 inhibitors alone are very well tolerated in patients with neutropenia being the most prominent on-target side effect^33,35,37^. MCL-1 inhibitors are currently being evaluated in early phase clinical trials and the toxicity profiles are unclear. We also demonstrated that concurrent inhibition of both BCL-2 and MCL-1 with low doses of BH3 mimetics was synergistic and overcame the heterogeneity in patient responses to single agent therapy. While the potential toxicities that may arise when these agents are used in combination are currently unknown, recent studies have sought to optimize concurrent therapy with these agents in murine models and demonstrated tolerability and efficacy in controlling acute myeloid leukemias^69^.

To maximize the therapeutic potential of these apoptosis-sensitizing agents, BH3 mimetics may be used in combination with other drugs that induce apoptotic signaling, such as proteasome inhibitors. Interestingly, when we combined BH3 mimetics with bortezomib or ixazomib, we found that the BCL-2 targeting agents ABT-199 and ABT-263 potently increased apoptosis in clonal plasma cells while combining proteasome inhibitors with the MCL-1 inhibitor S63845 did not. This contrasted sharply with the potent single-agent activity of S63845. We hypothesized that this may be due to changes in MCL-1 expression levels due to typical turnover of this very short-lived protein^70^ being mediated by the target of bortezomib: the 26 S proteasome. Instead, during bortezomib or ixazomib treatment, while we did not observe any changes in the expression of MCL-1, we found that all four of the patient samples that we tested exhibited an increase in the expression of Noxa. Noxa is an endogenous specific inhibitor of MCL-1, and its overexpression would naturally inhibit the pro-survival activity of MCL-1. Noxa does not detectably bind other pro-survival proteins, and thus targeting MCL-1 with S63845 in bortezomib-treated cells has no added benefit (Fig. S3a, b for model). Indeed, we detected potent upregulation of Noxa and its binding to MCL-1 upon bortezomib treatment, supporting our model. We also confirmed that this bortezomib-induced change in apoptotic dependencies could be detected directly in bone marrow aspirates from patients being actively treated. This suggests that analyzing specimens from patients on various therapies more broadly could uncover drug-induced apoptotic vulnerabilities in real-time that can be acted upon to improve outcomes.

A prominent challenge to the deployment of BH3 mimetics is accurately assigning therapies to patients when heterogeneity in responses is expected. We found that BH3 profiling was a strong predictor of ex vivo sensitivity of clonal plasma cells to specific BH3 mimetics, suggesting that it may be an effective tool for personalizing therapy. This is further aided by our observation that bortezomib and ixazomib sensitivity was correlated with overall apoptotic priming (response to the BIM BH3 peptide), suggesting that this assay may also help predict whether patients are likely to respond to proteasome inhibitors. We also found that t(11;14) positivity was associated with higher sensitivity to ABT-199 ex vivo, which is consistent with previous reports on increased BCL-2 dependence of multiple myeloma cells with this translocation^66,71^. Interestingly, t(11;14) was also associated with trending but not significant decreases in dependence on BCL-X_L_ and MCL-1; this aligns with previous studies showing higher BCL-2 gene and protein expression in myeloma cells with this translocation^72^. However, pronounced heterogeneity in the extent of BCL-2 dependence in t(11;14) AL amyloidosis and the clear BCL-2 dependence in some specimens lacking t(11;14) suggest that additional biomarkers may be particularly useful for assigning therapy with BH3 mimetics and especially ABT-199.

Our data suggest that BH3 mimetics are likely to be effective targeted treatments for AL amyloidosis, especially when deployed in tandem with a clinical biomarker that can inform treatment decisions. This is underscored by recent case reports and case series of patients treated with venetoclax-containing regimens that demonstrate deep yet heterogeneous responses^73–77^. Based on our results, highly primed clonal plasma cells from patients with AL amyloidosis would be sensitive to single agent treatment with a proteasome inhibitor, dexamethasone, or an inhibitor of MCL-1 or BCL-2, especially, when treatments are assigned with biomarkers such as BH3 profiling (Fig. S3). For patients with plasma cells that are more resistant to apoptosis, as identified by a functional assay such as BH3 profiling or protein expression and interaction analysis^78^, BCL-2 inhibitor treatment is more likely to be effective when used in combination with dexamethasone or a proteasome inhibitor. Indeed, previous studies have found that BH3 mimetics enhance myeloma sensitivity to dexamethasone^79–81^ or bortezomib^82,83^. It may also be possible to treat AL amyloidosis with these three agents in combination – such a strategy had promising efficacy and was adequately tolerated in early clinical trials for multiple myeloma^82^ and also substantially lengthened progression-free survival in the BELLINI trial (22.4 vs 11.5 months for ABT-199 vs placebo, respectively, in combination with bortezomib and dexamethasone)^84^. However, the ABT-199-treated patients also had increased rates of infection, which led to the premature termination of the trial. A trial evaluating venetoclax in combination with dexamethasone and the proteasome inhibitor ixazomib in AL amyloidosis patients with t(11;14) is currently underway (NCT04847453) and will provide insight into the efficacy and tolerability of this combination.

For patients with advanced disease that may not be able to tolerate bortezomib treatment at diagnosis due to excessive disease-induced organ dysfunction, use of an MCL-1 inhibitor as a single agent may be effective at eliminating those cells that would typically be sensitive to bortezomib while being less toxic to healthy tissues affected by amyloid deposition. If MCL-1 inhibition induced a reduction in clonal plasma cell populations and an improvement in organ function or patient performance status, initiation of combination therapy with a proteasome inhibitor and a BCL-2 inhibitor could then target remaining diseased cells. The similar proteomic alterations induced by bortezomib in both cell lines suggest that these baseline differences and especially BCL-2 family protein levels contribute to the blunted myeloma sensitivity to proteasome inhibition as well as dexamethasone and BH3 mimetics. A final key finding from our study is that the transformation of AL amyloidosis to symptomatic multiple myeloma at relapse may be associated with alterations in metabolism, cell cycle control and BCL-2 family proteins. These processes, and the pathways we found to be divergently induced, including MAPK, NF-κB and Wnt, could potentially be targeted for treatment. Our unbiased proteomic analysis exposed underlying differences between these diseases and suggests that AL-focused research may uncover novel therapeutic strategies for this disease that may not be present in multiple myeloma. The alterations in the abundance of metabolic pathway proteins are particularly interesting given that links have been established between electron transport chain activity and sensitivity to venetoclax in multiple myeloma^85^. Finally, we expect the proteomics data to be a valuable resource to AL amyloidosis and multiple myeloma researchers.

This work provides the first comprehensive characterization of apoptosis regulation in AL amyloidosis and identifies opportunities for targeting pro-survival protein dependencies in this disease – these may inform clinical trials design. Our findings also have implications for the clinical use of BH3 mimetics in other diseases that have reported apoptotic dependencies, including lung adenocarcinomas^38,86^, leukemias^71,87^, lymphomas^26,88^, and even certain central nervous system tumors^89^. Although there are significant challenges to overcome in the clinical deployment of these agents, especially the heterogeneity in apoptotic dependencies at baseline and during treatment with other therapies, as shown here, the early successes in therapeutically targeting pro-survival proteins are encouraging and show promise for treatment of AL amyloidosis.

## Methods

This project and all its experiments were completed under the approval of the Committee on Microbiological Safety of the Harvard T.H. Chan School of Public Health (HCSPH) Department of Environmental Health, protocol 17-245, and complies with all ethical regulations governed by said institution.

### Isolation of mononuclear cells

Bone marrow aspirates were collected from patients with treatment-naive and relapsed AL amyloidosis under protocol H-36533 at Boston Medical Center, which was approved by Panel Green Institutional Review Board (IRB) initially on 07/06/2017 and renewed annually for the duration of the studies. All patients provided statements of informed consent prior to sample collection. Mononuclear cells were isolated via Ficoll–Paque separation according to manufacturer’s instructions (GE Healthcare) and used for downstream applications.

### BH3 Profiling

For each sample, 8 × 10^6^ mononuclear cells were isolated, centrifuged at 2000 × g for 5 minutes, and resuspended in 150 µL FACS stain buffer (2% FBS in PBS). Cells were stained with conjugated cell-surface marker antibodies at 1:50 dilution: CD38 APC/Cy7 (102728, Biolegend, Dedham, MA) and CD138 Pacific Blue (356532, Biolegend). Cells were then centrifuged at 2000 × *g* for 5 minutes and subjected to BH3 Profiling as previously described^22^. After BH3 profiling, cells were permeabilized for intra-cellular staining with a saponin-based buffer (1% saponin, 10% BSA in PBS) and intracellular-staining antibodies for Cytochrome C AlexaFluor 647 (612310, Biolegend), Kappa Light Chain FITC (11-9970-42, Invitrogen), and Lambda Light Chain (12-9990-42, Invitrogen), used at 1:2000, 1:50, and 1:50 dilutions, respectively. Cells were left to stain overnight at 4 °C and analyzed by flow cytometry (Attune NxT) the following day. The %Specific Apoptosis (cytochrome c^-^) was calculated with the following equation (Eq. 1):

1 $$	\%{{{\rm{Specific}}}}\,{{{\rm{Apoptosis}}}}=\left[(\%{{{\rm{cyto}}}}\,{{{\rm{c}}}}\,{{{\rm{release}}}}\,[{{{\rm{Peptide}}}}])-\left(\%{{{\rm{cyto}}}}\,{{{\rm{c}}}}\,{{{\rm{release}}}}\right.\right.\\ 	\left.\left.[{{{\rm{Negative}}}}\,{{{\rm{Control}}}}]\right)\right]/\left[(\%{{{\rm{cyto}}}}\,{{{\rm{c}}}}\,{{{\rm{release}}}}\,[{{{\rm{Positive}}}}\,{{{\rm{Control}}}}])\right.\\ 	\left.-(\%{{{\rm{cyto}}}}\,{{{\rm{c}}}}\,{{{\rm{release}}}}\,[{{{\rm{Negative}}}}\,{{{\rm{Control}}}}])\right]$$

where “Negative Control” was either DMSO or PUMA2A (non-interacting mutated PUMA peptide)^22^ and “Positive Control” was BAX/BAK-independent pore-forming peptide Alamethicin^90^.

### Ex vivo chemosensitivity

Isolated mononuclear cells were plated at a concentration of 500,000 cells/mL in Dulbecco’s Modified Eagle Medium (11995065, Gibco) with 10% Fetal Bovine Serum and 1% Penicillin/Streptomycin and treated as indicated. Cells were collected at 24 and 48 h post-treatment and stained with antibodies for CD38 and CD138, and Alexa Fluor 647-conjugated Annexin V. Staining was fixed with 4% formaldehyde and 0.5% glutaraldehyde in 1x Annexin V binding buffer. After 10 minutes incubation, fixation was neutralized with N2 buffer (Tris Glycine). Cells were then stained for Κ/Lamba light chains as described above and analyzed by flow cytometry. Due to the propensity for plasma cells to lose expression of CD138 while undergoing apoptosis^78,91–93^, %Cell Death was calculated as (Eq. 2):

2 $$\%{{{{{\rm{Cell}}}}}}\,{{{{{\rm{death}}}}}}=(\%{{{{{{\rm{CD138}}}}}}}^{+}[{{{{{\rm{Drug}}}}}}])/(\%{{{{{{\rm{CD138}}}}}}}^{+}[{{{{{\rm{DMSO}}}}}}])$$

Specific Apoptosis (Annexin^+^) was calculated in CD138^+^ cells as (Eq. 3):

3 $$	\%{{{\rm{Specific}}}}\,{{{\rm{Apoptosis}}}}=[(\%{{{{\rm{Annexin}}}}}^{+}[{{{\rm{Drug}}}}])-(\%{{{{\rm{Annexin}}}}}^{+}[{{{\rm{DMSO}}}}])]\\ 	/[100-\big(\%{{{{\rm{Annexin}}}}}^{+}[{{{\rm{DMSO}}}}]]$$

### In vitro cell culture

ALMC-1 and ALMC-2 cell lines were cultured as previously described^54^. Diffuse large B cell lymphoma (SUDHL7 and OCI-Ly8) and multiple myeloma (AMO1, MM1S, HuNS, and RPMI-8226) cell lines were provided by Stephan Bohl from the laboratory of Anthony Letai at the Dana-Farber Cancer Institute. These cells were all cultured in RPMI (Invitrogen) in 10% FBS with 1% penicillin/streptomycin (Invitrogen).

### Cell line chemosensitivity assays

Cells were plated in 96-well plates, treated for with drugs at indicated doses, then collected at 12 or 24 h post treatment. Cells were then stained with Alexa Fluor 647-conjugated Annexin V in 10x Annexin V binding buffer, diluted 1/10 into cell solution. Annexin V positivity was then measured by flow cytometry with Attune NxT flow cytometer (Thermo Fisher).

### Cell line siRNA knockdown

ALMC-1 and ALMC-2 cells were plated in 6-well plates at 1 × 10^6^cells/well. Eighty-one microliters of Lipofectamine RNAiMAX (ThermoFisher Scientific) was diluted into 900 µL Opti-MEM Medium (ThermoFisher). Dilutions of siRNA were prepared by adding 6 µL of the following 10 µM siRNA solutions into separate 300 µL Opti-MEM Medium: Noxa, Bim, and control siRNA (Santa Cruz Biotechnologies, sc-37305, sc-29802, and sc-37007). The diluted siRNA solutions were then added to the diluted Lipofectamine solutions at a 1:1 ratio and incubated for 5 minutes at room temperature. 250 µL of each siRNA-lipid complex solution was added to a well of the 6-well plate. Cells were transfected for 24 h at 37 °C, then plated onto 96 well plates and treated with drugs at indicated doses. Viability was then measured after 12 hours post treatment by Annexin V assay described previously.

### Statistics and reproducibility

One-way, Two-way ANOVA, Spearman rho correlation and paired *t* tests were performed using the GraphPad Prism software (GraphPad Software). Significance: *p < 0.05; **p < 0.01; ***p < 0.001; ****p < 0.0001. Linear regression and principal components analysis were performed on log2-transformed mass spectrometry data standardized to have approximately zero mean and common variance for each row and column. Covariates in the linear regression model consisted of a dummy variable for cell line (ALMC-1 versus ALMC-2), treatment duration in hours, and interaction between cell line and treatment duration. Adjusted p-values were computed using the Bonferroni correction. Western blots from both primary patient samples and cell lines and mass spectrometry were performed once. All other experiments were performed at least thrice unless otherwise indicated. Statistical tests used for each experiment are indicated on figures and were always two-sided. “ns” designates “not significant” on graphs.

### Whole cell proteomics: Cell lysis, protein digest and TMT labeling mass spectrometry analysis

For each time point studied (0, 2, 4, 6, and 12 h), a single sample corresponding to approximately 1 × 10^7^ cells was treated with 10 nM bortezomib, collected, washed with PBS and lysed by homogenization (QIAshredder cartidges, Qiagen) in lysis buffer (2% SDS, 150 mM NaCl, 50 mM Tris pH 7.4). Reductive alkylation was then performed with 5 mM DTT for 30 min at 37 °C followed by alkylation using 0.4 M iodoacetamide in 50 mM ammonium bicarbonate, to a final concentration of 25 mM iodoacetamide for 30 minutes in the dark. Alkylation reactions were quenched with freshly prepared DTT added to a concentration of 50 mM and proteins were precipitated by methanol/chloroform precipitation. Digests were carried out in 1 M urea freshly prepared in 200 mM EPPS pH 8.5 in the presence of 2% acetonitrile (v/v) with LysC (Wako, 2 mg/ml, used 1:75 w/w protease: substrates during digest) for 3 hours at room temperature and after subsequent addition of trypsin (Promega #V5111, stock 1:100 w/w protease: substrates) over night at 37 °C. The rate of missed cleavages was assayed by mass spectrometry. For proteomic analysis, digests containing approximately 60 μg of peptide material were directly labeled with TMT 11plex reagents (Thermo Fisher Scientific). Labeling efficiency and TMT ratios were assayed by mass spectrometry. Following quenching of TMT labeling reactions with with a final concentration of 0.5% hydroxylamine, TMT labelled peptides were mixed, solvent evaporated and TMT labeled peptides purified and desalted by acidic reversed phase C18 chromatography. Peptides were then fractionated by alkaline reversed phase chromatography into 96 fractions and combined into 24 fractions^94^.

### Mass spectrometry analysis

Data were collected using a MultiNotch MS3 TMT method^95^ using an Orbitrap Lumos mass spectrometer coupled to a Proxeon EASY-nLC 1200 liquid chromatography (LC) system (both Thermo Fisher Scientific). Column and matrix used were 35 cm column length, 75 μm inner diameter, matrix 2.6 μm Accucore (Thermo Fisher Scientific). Peptides of each fraction were separated with 4 h acidic acetonitrile gradients by LC prior to mass spectrometric (MS) analysis. MS1 scans (Orbitrap analysis; resolution 120,000; mass range 400–1400 Th) were followed by MS2 analysis with collision-induced dissociation (CID, CE = 35) and a maximum ion injection time of up to 120 ms and an isolation window of 0.4 m/z. In order to obtain quantitative information, MS3 precursors were fragmented by high-energy collision-induced dissociation (HCD) and analyzed in the Orbitrap at a resolution of 50,000 at 200 Th. Further details on LC and MS parameters and settings used were described recently^96^.

Peptides were searched with a SEQUEST (v.28, rev. 12) based software against a size-sorted forward and reverse database of the H. sapiens proteome (Uniprot 07/2014) with added common contaminant proteins. Searches were performed using a mass tolerance of 20 ppm for precursors and a fragment ion tolerance of 0.9 Da. For the searches maximally 2 missed cleavages per peptide were allowed. Oxidized methionine residues (+15.9949 Da) were searched dynamically. A target decoy database strategy was applied, and a false discovery rate (FDR) of 1% was set for peptide-spectrum matches following filtering by linear discriminant analysis (LDA). The FDR for final collapsed proteins was 1%. MS1 data were calibrated post search, and searches were performed again. Quantitative information on peptides was derived from MS3 spectra. Quant tables were filtered for an MS2 isolation specificity of >70% for each peptide and a sum of TMT signal to noise (s/n) of >200 over all channels. Details of the TMT intensity quantification method and further search parameters applied were described previously^96^. Proteomics raw data and search results were deposited in the PRIDE archive and can be accessed under ProteomeXchange accession number: PXD024119.

Bioinformatic analyses were performed using the R statistical computing environment (http://www.r-project.org, version 4.1.2). Heatmaps were constructed with the ComplexHeatmap package, using the complete-linkage method based on Euclidean distance, and sub-clusters were separated by k means partitioning. Gene set enrichment analysis was performed with the clusterProfiler package. Analysis was performed for all three subontologies (“BP”, “MF” and “CC”) using minimal and maximal sizes of each geneSet of 3 and 5000, respectively, and a BH-adjusted *p* value cutoff of 0.05.

### Immunoblotting

Immunoblotting was performed as previously described^45^. Briefly, cells were lysed using Radio Immuno Precipitation Assay (RIPA) Buffer (New England Biolabs) with protease inhibitor cocktail (Roche). Protein loading was measured by Bradford reagent (NEB). Samples were separated by gel electrophoresis on Bio-Rad AnyKD gels. Antibodies used at indicated dilutions: Anti-Noxa (114C307) (OP180, Millipore Sigma) at 1:2000, Anti-BAX (2772, Cell Signaling Technology) at 1:4000, Anti-BCL-xL (2764, Cell Signaling Technology) at 1:4000, Anti-GAPDH (2118, Cell Signaling Technology) at 1:8000, Anti-MCL1 rAb (94296, Cell Signaling Technology) at 1:2000, Anti-BCL-2 (2872, Cell Signaling Technology) at 1:2000, Anti-BIM (2933, Cell Signaling Technology) at 1:4000, Anti-ATF4 (11815, Cell Signaling Technology) at 1:4000, Anti-CHOP (2895, Cell Signaling Technology) at 1:4000.

### Immunoprecipitation

Immunoblotting was performed per antibody manufacturer’s specifications. Cells were lysed using Tris Lysis Buffer and Protease inhibitor cocktail (Meso Scale Diagnostics) and immunoprecipitated overnight at 4 °C with Protein A/G Agarose beads (Thermo Fisher) and the following antibodies: anti-MCL1 Clone 22 (559027, BD Pharmingen) at 1:500, anti-BIM (2819, Cell Signaling Technology) at 1:200, anti-BCL-2 (15071, Cell Signaling Technology) at 1:200, and anti-mouse IgG1 (G3A1) (5415, Cell Signaling Technology) at 1μg/100μg protein. The immunoprecipitate and a sample of the initial whole-cell lysate for each condition were then analyzed by western blot.

### Mouse housing and ALMC-1 xenograft model

Animal experiments were performed under Harvard Institutional Animal Care and Use Committee protocol IS00001059 following ethical approval and NIH guidelines for animal welfare were followed. Mice were housed in 50% humidity at 70°F ambient temperature and exposed to room lighting from 7 am to 7 pm. Animals were housed and cared for according to standard guidelines with free access to water and food. All experiments were performed on 8-16 week old NOD-*scid* IL2Rgamma^null^ NSG mice (Jackson labs stock#005557) of both sexes. Animals were randomly assigned to experimental groups. Mice were injected with 10^6^ cells in matrigel subcutaneously. Mice were monitored daily for tumor growth and treatment was initiated at a tumor volume of 150 mm^3^, with treatment subsequently lasting 4 weeks. Venetoclax cohort of mice were treated by oral gavage daily with ABT-199 (Venetoclax) (Medchem express) at a dose of 100 mg/kg in a mixture of 60% phosal 50 PG, 30% PEG 400, and 10% EtOH. Bortezomib cohort were treated every other day by IP Bortezomib (Medchem express) injection at a dose of 0.8 mg/kg in 0.9% saline. Vehicle treated mice received both vehicles. Tumor growth was monitored every other day.

### Quantitative PCR (qPCR)

mRNA was isolated using the Qiagen RNeasy mini kit (Qiagen 74104). Isolated and equilibrated mRNA was reverse transcribed using Thermo Fisher High-Capacity cDNA Reverse Transcription Kit (Thermo 4368814). qPCR was run in 96 well plate format using BIO RAD SYBR Green Master Mix (BIO RAD 1725270). All qPCR experiments were run in triplicate and normalized using primers targeting Human GAPDH (F: 5’ -GCACCGTCAAGGCTGAGAAC- 3’ R: 5’ -TGGTGAAGACGCCAGTGG A- 3’). The following primer pairs were used for detection of their respective targets: PMAIP1 (5’ -F:CTGGAAGTCGAGTGTGCTACTC- 3’ R: 5’ -TGAAGGAGTCCCCTCATGCAAG- 3’), Bim (F: 5’ -GGAGACGAGTTTAACGCTTAC- 3’ R: 5’ -CAAGCAAAATGTCTGCATGG- 3’), BCL-2 (F: 5’ -ATCGCCCTGTGGATGACTGAGT- 3’ R: 5’ -GCCAGGAGAAATCAAACAGAGGC- 3’), BAX (F: 5’ -TGGAGCTGCAGAGGATGATTG- 3’ R: 5’ -GAAGTTGCCGTCAGAAAACATG- 3’), BAK (F: 5’ - TGAGTACTTCACCAAGATTGCCA- 3’ R: 5’ -AGTCAGGCCATGCTGGTAGAC- 3’), MCL-1 (F: 5’ -GTAATAACACCAGTACGGACGG- 3’ R: 5’ -TCCCGAAGGTACCGAGAGAT).

### Seahorse XF mito stress test

ALMC-1 and ALMC-2 cells were plated at 140,000 cells/well in a 96 well Seahorse XF microplate (Agilent) coated with Cell-TAK cell adhesive (354249, Corning). Cells were subjected to Mito Stress test per manufacturer’s instructions (Agilent). Microplate was inserted into the XF96, and OCR was measured every 5 minutes over the course of the assay, with mixing and waiting time in between measurements. In 15 minute increments, the following reagents were added to each well at indicated concentrations: 1.5 μM oligomycin, 1 μM FCCP, 0.5 μM rotenone/antimycin A mixture.

### Reporting summary

Further information on research design is available in the Nature Research Reporting Summary linked to this article.

## Supplementary information

Supplementary Information

Description of Additional Supplementary Files

Supplementary Dataset 1

Reporting Summary

## Data Availability

The mass spectrometry data that support the findings of this study are available in the PRIDE database: https://www.ebi.ac.uk/pride/ ProteomeXchange accession number: PXD024119 The authors declare that all other source data supporting the findings of this study are available within the paper and its supplementary information files, including the Source Data file. Source data are provided with this paper.

## Source data

Source Data
