# Supplementary material for: Exploiting endogenous and therapy-induced apoptotic vulnerabilities in immunoglobulin light chain amyloidosis with BH3 mimetics
Source: Nat Commun. 2022 Oct 2;13:5789. doi: 10.1038/s41467-022-33461-z (PMC9527241; doi:10.1038/s41467-022-33461-z)
Supplement: Supplementary file 1 — Supplementary Information [file 41467_2022_33461_MOESM1_ESM.pdf]

Supplementary Information

Exploiting endogenous and therapy-induced apoptotic vulnerabilities in immunoglobulin light chain amyloidosis with BH3 mimetics

Cameron S. Fraser[1,2,3], Johan K.E. Spetz[1,2,3], Xingping Qin[1,2,3], Adam Presser[1,2,3], Jonathan Choiniere[1,2,3], Chendi Li[4,5], Stacey Yu[1,2,3], Frances Blevins[6,7], Aaron N. Hata[4,5], Jeffrey W. Miller[8], Gary A. Bradshaw[3], Marian Kalocsay[3,9], Vaishali Sanchorawala[6,7], Shayna Sarosiek[6,7,10,#], Kristopher A. Sarosiek[1,2,3,#]

John B. Little Center for Radiation Sciences, Harvard TH Chan School of Public Health, Boston, MA 02115, USA
[2]Program in Molecular and Integrative Physiological Sciences, Harvard TH Chan School of Public Health, Boston, MA 02115, USA

[3]Laboratory of Systems Pharmacology, Harvard Medical School, Boston, MA 02115, USA
[4]Massachusetts General Hospital Cancer Center, Charlestown, MA 02129, USA
[5]Department of Medicine, Harvard Medical School, Boston, MA 02115, USA
[6]Section of Hematology & Medical Oncology, Boston Medical Center, Boston, MA 02118, USA
[7]Amyloidosis Center, Boston University School of Medicine, Boston, MA 02118, USA
[8]Department of Biostatistics, Harvard TH Chan School of Public Health, Boston, MA 02115, USA
[9]Present address: Department of Experimental Radiation Oncology, University of Texas MD Anderson Cancer Center, Houston, TX 77030, USA

[10]Present address: Dana-Farber Cancer Institute, Harvard Cancer Center, Boston, MA 02215, USA
[#]Co-corresponding authors

For correspondence:

Kristopher Sarosiek, Ph.D. (sarosiek@hsph.harvard.edu)

Shayna R. Sarosiek, M.D. (Shayna_sarosiek@dfci.harvard.edu)

Fraser et. al

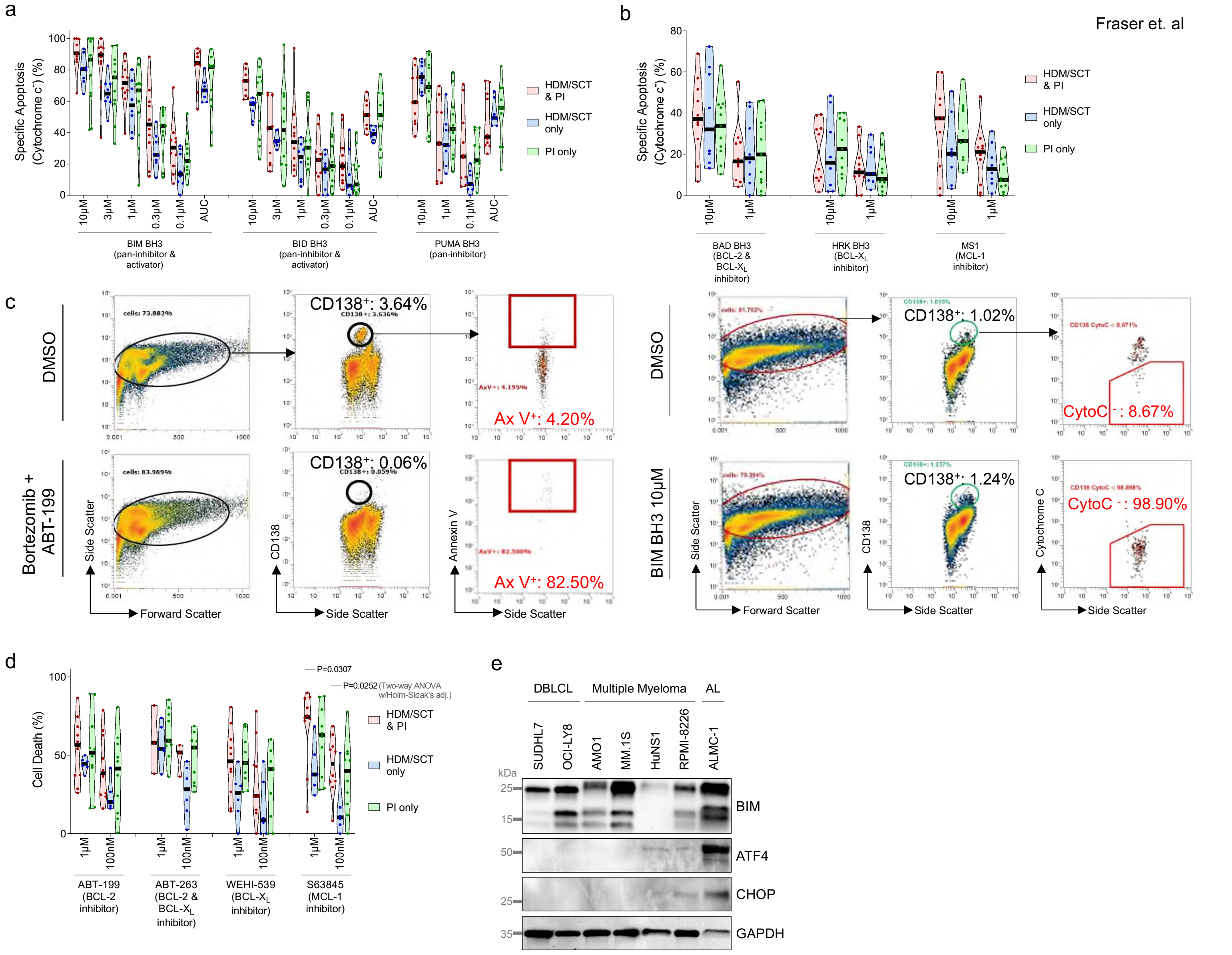

Supplementary Figure 1: Flow cytometry analysis of primary AL amyloidosis specimens and analysis based on prior therapy. (a-b) BH3 profiling measurement of overall priming (a) and apoptotic dependencies (b) based on type of prior therapy patients received. (c) Representative flow cytometry plots for identifying plasma cells (CD138⁺) that undergo cell death, as measured by reduction in plasma cell percentage of total bone marrow mononuclear cells, in response to chemotherapeutic treatment, and representative BH3 profiling flow cytometry plots showing gating strategy for identifying Cytochrome C retention or loss in response to peptide treatment of plasma cells (CD138⁺). (d) AL amyloidosis clonal plasma cell sensitivity to BH3 mimetics based on type of prior therapy patients received. P-values were calculated using two-way ANOVA with Holm-Sidak's adj.(e) Comparison of BIM, ATF4 and CHOP levels in diffuse large B cell lymphoma, multiple myeloma and ALMC-1 cell lines.

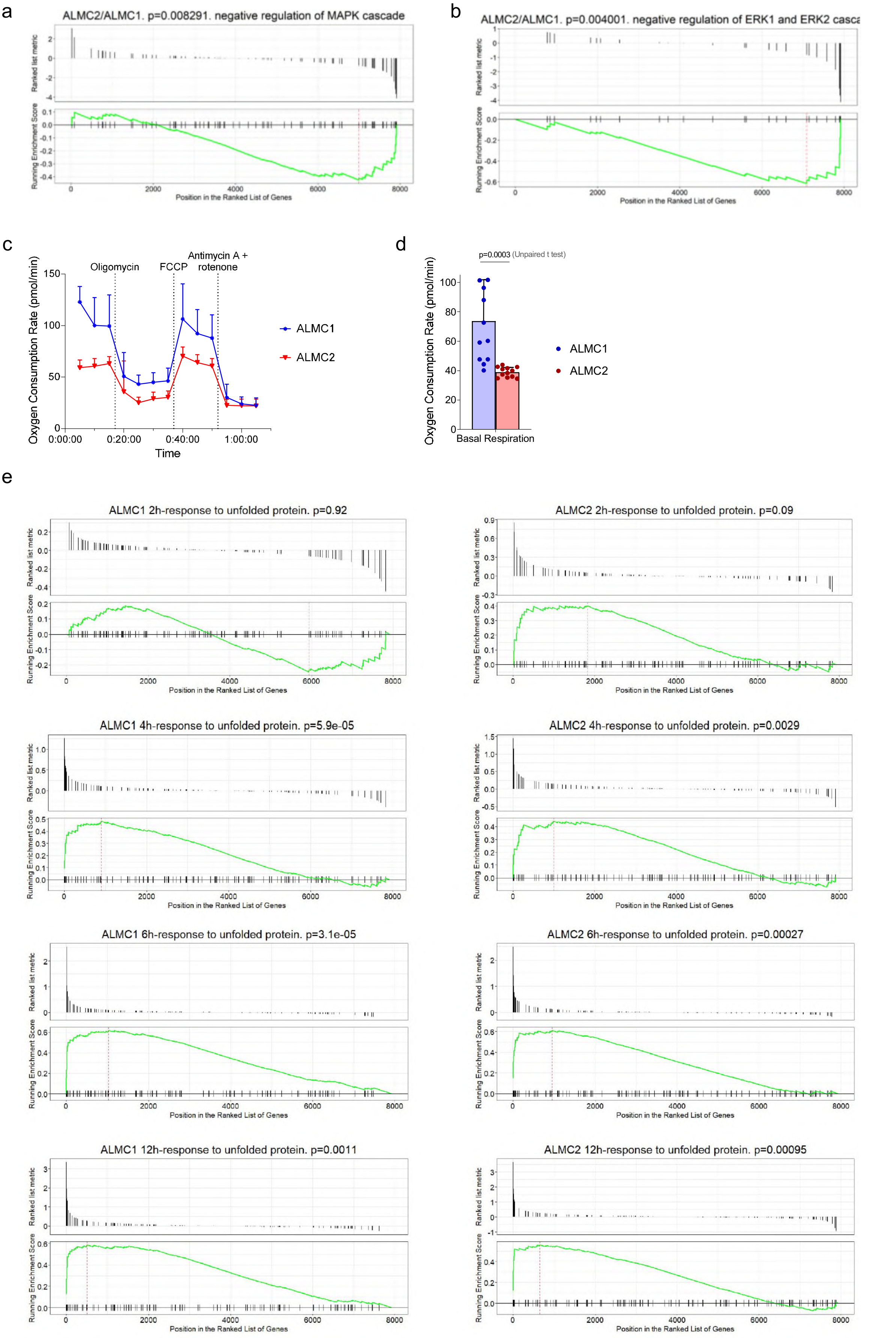

Supplementary Figure 2. Mass spectrometry analysis of bortezomib-treated ALMC-1 and ALMC-2 cells. (a-b) Gene set enrichment analysis for negative regulation of (a) MAPK signaling and (B) ERK1 and ERK2 signaling. (c) Seahorse analysis of ALMC-1 and ALMC-2 cells demonstrating increased mitochondrial respiration at baseline (before addition of oligomycin). (n=2) (d) Quantification of oxygen consumption by basal mitochondrial respiration of ALMC-1 and ALMC-2 cells. Data are presented as mean values (n=2) +/- StDev. P-values were calculated using unpaired *t* test. (e). Enrichment analysis for proteins (genes) associated with unfolded protein response compared with untreated (time 0 hr) cells.

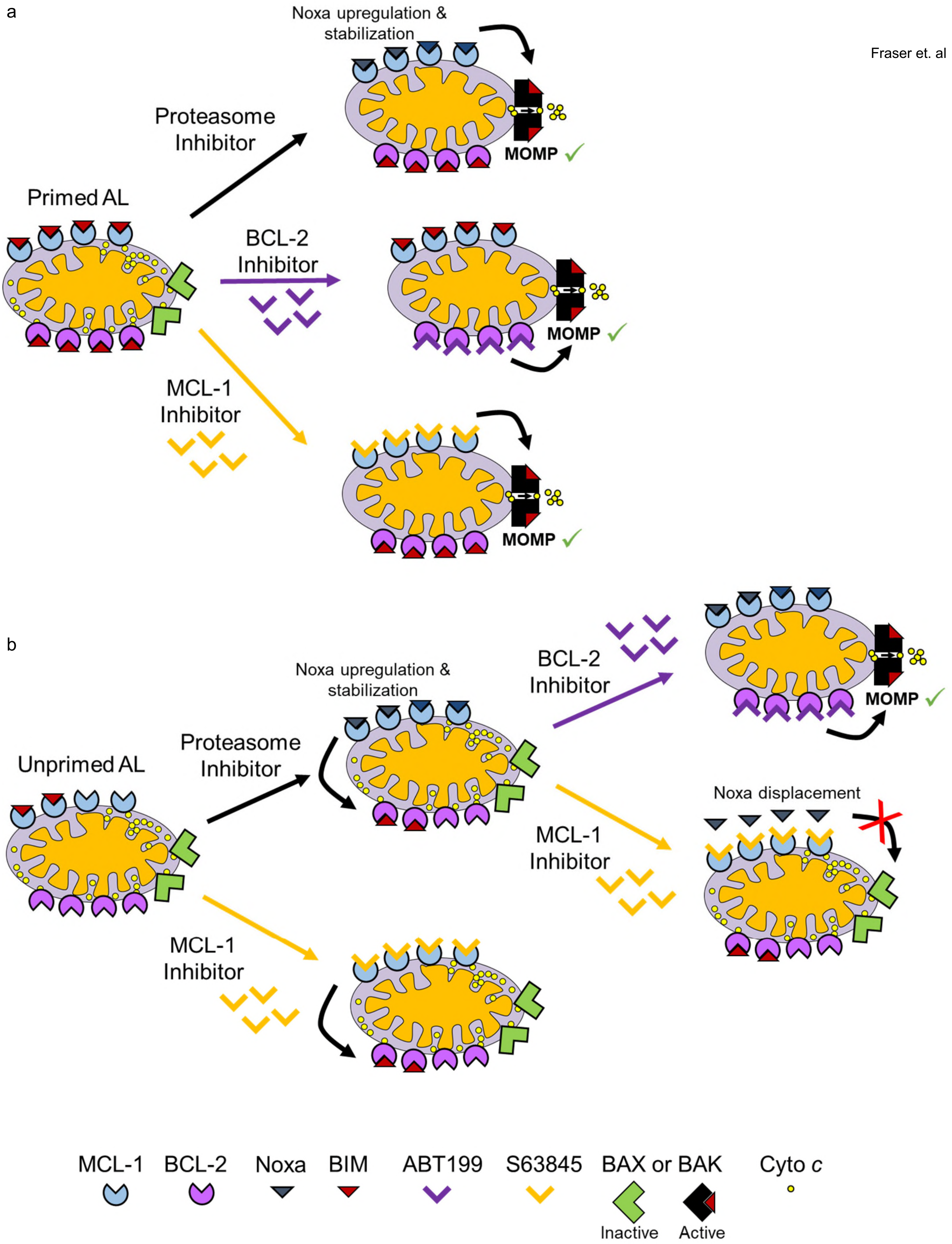

Supplementary Figure 3. Exploiting apoptotic vulnerabilities in AL amyloidosis. Model for changes in BCL-2 family dependencies in plasma cells from AL amyloidosis. (a) Apoptosis can be induced in highly primed AL clonal plasma cells by 1) treating with a proteasome inhibitor that stabilizes and upregulates the endogenous MCL-1 inhibitor Noxa; 2) treating with a small molecular MCL-1 inhibitor; or 3) treating with a BCL-2 inhibitor. (b) Unprimed clonal plasma cells do not undergo apoptosis in response to proteasome, BCL-2 or MCL-1 inhibition. However, treatment with a proteasome inhibitor stabilizes and upregulates Noxa and results in BCL-2 dependence, which can be exploited via BCL-2 inhibition. Note that MCL-1 inhibition does not induce apoptosis in proteasome inhibited cells since Noxa does not efficiently activate BAX or BAK.
