# Supplementary material for: Exploiting endogenous and therapy-induced apoptotic vulnerabilities in immunoglobulin light chain amyloidosis with BH3 mimetics
Source: Nat Commun. 2022 Oct 2;13:5789. doi: 10.1038/s41467-022-33461-z (PMC9527241; doi:10.1038/s41467-022-33461-z)
Supplement: Supplementary file 2 — Description of Additional Supplementary Files [file 41467_2022_33461_MOESM2_ESM.docx]

**Description of Additional Supplementary Files**

**Supplementary Dataset 1:** Baseline patient characteristics. Organ involvement key: ANS = autonomic nervous system; PNS = peripheral nervous system; GI = gastrointestinal. Treatment key: C = cyclophosphamide; D = dexamethasone; Dara = Daratumumab; HDM/SCT= high dose melphalan with stem cell transplantation; I = Ixazomib; P = pomalidomide; R = lenalidomide; Ritux = Rituximab; V = velcade (bortezomib).
