# Supplementary material for: Exploiting endogenous and therapy-induced apoptotic vulnerabilities in immunoglobulin light chain amyloidosis with BH3 mimetics
Source: Nat Commun. 2022 Oct 2;13:5789. doi: 10.1038/s41467-022-33461-z (PMC9527241; doi:10.1038/s41467-022-33461-z)
Supplement: Supplementary file 4 — Reporting Summary [file 41467_2022_33461_MOESM4_ESM.pdf]

nature portfolio

Corresponding author(s): Kristopher Sarosiek; Shayna Sarosiek

Last updated by author(s): 8/20/2022

Reporting Summary

Nature Portfolio wishes to improve the reproducibility of the work that we publish. This form provides structure for consistency and transparency in reporting. For further information on Nature Portfolio policies, see our Editorial Policies and the Editorial Policy Checklist.

Statistics

For all statistical analyses, confirm that the following items are present in the figure legend, table legend, main text, or Methods section.

n/a	Confirmed	
☐	☒	The exact sample size (*n*) for each experimental group/condition, given as a discrete number and unit of measurement
☐	☒	A statement on whether measurements were taken from distinct samples or whether the same sample was measured repeatedly
☐	☒	The statistical test(s) used AND whether they are one- or two-sided<br>*Only common tests should be described solely by name; describe more complex techniques in the Methods section.*
☐	☒	A description of all covariates tested
☐	☒	A description of any assumptions or corrections, such as tests of normality and adjustment for multiple comparisons
☐	☒	A full description of the statistical parameters including central tendency (e.g. means) or other basic estimates (e.g. regression coefficient) AND variation (e.g. standard deviation) or associated estimates of uncertainty (e.g. confidence intervals)
☐	☒	For null hypothesis testing, the test statistic (e.g. *F*, *t*, *r*) with confidence intervals, effect sizes, degrees of freedom and *P* value noted<br>*Give P values as exact values whenever suitable.*
☐	☒	For Bayesian analysis, information on the choice of priors and Markov chain Monte Carlo settings
☐	☒	For hierarchical and complex designs, identification of the appropriate level for tests and full reporting of outcomes
☐	☒	Estimates of effect sizes (e.g. Cohen's *d*, Pearson's *r*), indicating how they were calculated

Our web collection on statistics for biologists contains articles on many of the points above.

Software and code

Policy information about availability of computer code

Data collection: No software was used for data collection

Data analysis: Graphpad Prism (9.3.1) was used for figure production and data/statistical analysis. Bioinformatic analyses were performed using the R statistical computing environment (http://www.r-project.org, version 4.1.2), specific packages outlined in Methods.

For manuscripts utilizing custom algorithms or software that are central to the research but not yet described in published literature, software must be made available to editors and reviewers. We strongly encourage code deposition in a community repository (e.g. GitHub). See the Nature Portfolio guidelines for submitting code & software for further information.

Data

Policy information about availability of data

All manuscripts must include a data availability statement. This statement should provide the following information, where applicable:

- Accession codes, unique identifiers, or web links for publicly available datasets
- A description of any restrictions on data availability
- For clinical datasets or third party data, please ensure that the statement adheres to our policy

The mass spectrometry data that support the findings of this study will be available in the PRIDE database:

https://www.ebi.ac.uk/pride/
ProteomeXchange accession number: PXD024119
Username: reviewer_pxd024119@ebi.ac.uk
Password: yDU0nLxX

The authors declare that all other data supporting the findings of this study are available within the paper and its supplementary information files.

Field-specific reporting

Please select the one below that is the best fit for your research. If you are not sure, read the appropriate sections before making your selection.

☒ Life sciences ☐ Behavioural & social sciences ☐ Ecological, evolutionary & environmental sciences

For a reference copy of the document with all sections, see nature.com/documents/nr-reporting-summary-flat.pdf

Life sciences study design

All studies must disclose on these points even when the disclosure is negative.

Sample size	Our sample size was limited by an agreement with Boston Medical Center for this collaborative project. We were provided 95 samples, sufficient for comprehensive and thorough analysis.
Data exclusions	Patient sample data was excluded when total viable cell abundance, measured at the time of sample collection, was insufficient for comprehensive analysis (under 1.6x10^7 cells), or later excluded when %CD138 positive cells measured by flow cytometry was under 1% of total cells, indicating insufficient clonal cell abundance for analysis.
Replication	When possible, experiments were run in triplicate to confirm reproducibility. All in vitro cell line drug treatments were performed in triplicate. Many of the experiments were run with primary patient biopsies, so we were limited in the material available for a single patient to perform technical replicates. Instead, multiple patient samples were used for the same experiments, providing biological replicates. In vitro drug sensitivity cell line experiments were completed in triplicate and replicate results were highly similar. Western blots of primary samples were performed once due to limited sample availability, but several patient samples were analyzed in a similar manner yielding similar results. Western blots of cell lines were run once but results were confirmed by proteomic analysis. Xenograft experiments were performed with n=5 in each treatment group. For flow cytometry experiments, identical gating was applied across experiments to eliminate bias.
Randomization	Mice bearing AL amyloidosis cell line xenografts were randomly assigned to each group for treatment. Patient samples were randomly assigned into different experiments and experimental groups as the disease status was not known to investigators until after sample collection and analysis.
Blinding	Investigators were blinded as to the treatment/disease status of the patient at the time of sample collection, only afterwards were patient samples binned into relevant cohorts for the study. For in vitro drug sensitivity, the experimental operator was blinded as to the sample status, but blinding related to in vitro treatment was not possible as experiments were performed by a single operator. For subsequent flow cytometry analysis, identical gating was applied across experiments and across samples within an experiments to eliminate bias.

Reporting for specific materials, systems and methods

We require information from authors about some types of materials, experimental systems and methods used in many studies. Here, indicate whether each material, system or method listed is relevant to your study. If you are not sure if a list item applies to your research, read the appropriate section before selecting a response.

Materials & experimental systems

n/a	Involved in the study	
☐	☒	Antibodies
☐	☒	Eukaryotic cell lines
☒	☐	Palaeontology and archaeology
☐	☒	Animals and other organisms
☐	☒	Human research participants
☒	☐	Clinical data
☐	☒	Dual use research of concern

Methods

n/a	Involved in the study	
☒	☐	ChIP-seq
☐	☒	Flow cytometry
☒	☐	MRI-based neuroimaging

Antibodies

Antibodies used

Western blotting:
Anti-Noxa (114C307) (OP180, Millipore Sigma)
Anti-BAX (2772, Cell Signaling Technology)
Anti-BCL-xL (2764, Cell Signaling Technology)
Anti-GAPDH (2118, Cell Signaling Technology)
Anti-MCL1 rAb (94296, Cell Signaling Technology)
Anti-BCL-2 (2872, Cell Signaling Technology)
Anti-BIM (2933, Cell Signaling Technology)
Anti-ATF4 (11815, Cell Signaling Technology)
Anti-CHOP (2895, Cell Signaling Technology)

Immunoprecipitation:
Anti-BIM (2819, Cell Signaling Technology)
Anti-BCL-2 (15071, Cell Signaling Technology)
Anti-MCL-1 Clone 22 (559027, BD Biosciences)

Flow cytometry:
Alexa Fluor® 647 anti-Cytochrome c clone 6H2.B4 (612310, Biolegend)
Pacific Blue™ anti-human CD138 (Syndecan-1) clone MI15 (356532, Biolegend)
APC/Cyanine7 anti-human CD38 clone HIT2 (303534, Biolegend)

Validation

-Anti-BAX (2772, Cell Signaling Technology) detects endogenous levels of total Bax protein. The antibody does not cross-react with other Bcl-2 family members. Species Reactivity: Human, Mouse, Rat, Monkey. Application: WB, IP.
-Anti-BCL-xL (2764, Cell Signaling Technology) detects endogenous levels of total Bcl-xL protein. The antibody does not cross-react with other Bcl-2 family members. Species Reactivity: Human, Mouse, Rat, Monkey. Application: WB, IP, IHC, IF, FC. Monoclonal antibody is produced by immunizing animals with a synthetic peptide corresponding to residues surrounding Asp61 of human Bcl-xL.
-Anti-GAPDH (14C10) (2118, Cell Signaling Technology) detects endogenous levels of total GAPDH protein. Species Reactivity: Human, Mouse, Rat, Monkey, Bovine, Pig. Application: WB, IHC, IF, FC. Monoclonal antibody is produced by immunizing animals with a synthetic peptide near the carboxy terminus of human GAPDH.
-Anti-MCL-1 (D2W9E) (94296, Cell Signaling Technology) recognizes endogenous levels of total Mcl-1 protein. Monoclonal antibody is produced by immunizing animals with a synthetic peptide corresponding to residues surrounding Pro60 of mouse Mcl-1 protein. Species Reactivity: Human, Mouse, Rat. Applications: WB, IP, IHC, FC.
-Anti-BCL2 antibody(2872, Cell Signaling Technology) detects endogenous levels of total Bcl-2 protein. The antibody does not cross-react with other Bcl-2 family members. Produced by immunizing animals with a synthetic peptide corresponding to residues surrounding Gly41 of human Bcl-2, a region not conserved in mouse or rat. Purified by protein A and peptide affinity chromatography. Application: WB. Species Reactivity:
Human
-Anti-BIM antibody (C34C5) (2933, Cell Signaling Technology) detects endogenous levels of total Bim (EL, L and S isoforms) protein. Produced by immunizing animals with a synthetic peptide corresponding to residues surrounding Pro25 of Bim. Species Reactivity: Human, Mouse, Rat. Application: WB, IP, IHC, IF, FC.
-Anti-ATF4 antibody (D4B8) (11815, Cell Signaling Technology) recognizes endogenous levels of total ATF-4 protein. Species Reactivity: Human, Mouse, Rat. Monoclonal antibody is produced by immunizing animals with a synthetic peptide corresponding to residues near the carboxy terminus of human ATF-4 protein. Applications: WB, IP, IF, ChIP, ChIP-seq.
-Anti-CHOP antibody (L63F7) (2895, Cell Signaling Technology) detects endogenous levels of total CHOP protein. Species Reactivity: Human, Mouse, Rat. Monoclonal antibody is produced by immunizing animals with a synthetic peptide corresponding to the sequence of human CHOP. Applications: WB, IP, IF, FC, ChIP
-Anti-BIM antibody (2819, Cell Signaling Technology) detects endogenous levels of total Bim (EL, L and S isoforms) protein. Species Reactivity: Human, Mouse, Rat, Monkey. Antibody produced by immunizing animals with a synthetic peptide corresponding to residues near the amino terminus of human Bim and purified by protein A and peptide affinity chromatography. Applications: WB, IP.
-Anti-BCL-2 antibody (124) (15071, Cell Signaling Technology) recognizes endogenous levels of total Bcl-2 protein. Monoclonal antibody is produced by immunizing animals with a synthetic peptide corresponding to residues surrounding Gly47 of human Bcl-2 protein. Species Reactivity:
Human. Applications: WB, IP, IHC, FC.
-Anti-MCL-1 antibody (Clone 22) (559027, BD Biosciences) detects endogenous levels of human Mcl-1. The monoclonal antibody purified from tissue culture supernatant or ascites by affinity chromatography. The antibody is tested by Western blot on K-562 cell lysate (ATCC CCL-243). Applications: WB blot (Routinely Tested), IP (Tested During Development)
-Anti-Noxa antibody (114C307) (OP180, Millipore Sigma) is validated for use in Immunoblotting for the detection of human Noxa protein. The antibody is tested by Western blot on RL-7 cell lysate. Species reactivity: Human. Application: WB. Monoclonal antibody produced with human Noxa fused to GST immunogen.
-Anti-cytochrome C antibody (6H2.B4) (612310, Biolegend) recognizes human, mouse, and rat cytochrome-c. The antibody was purified by affinity chromatography and conjugated with Alexa Fluor® 647 under optimal conditions. Has been validated for use in intracellular flow cytometric staining, Western blotting, immunoprecipitation, and immunofluorescence staining. Each lot is quality control tested by immunofluorescent staining with flow cytometric analysis.
-Anti CD138 Syndecan-1 antibody (MI15) (356532, Biolegend) recognizes human CD138 (Syndecan-1). Monoclonal antibody was produced by a mixture of U266 and XG-1 human myeloma cell line immunogens. Has been validated for use in flow cytometry. Each lot is quality control tested by immunofluorescent staining with flow cytometric analysis.
-Anti CD38 antibody (HIT2) (303534, Biolegend) recognizes human, chimpanzee, horse, and cow CD38. The antibody was purified by affinity chromatography and conjugated with APC/Cyanine7 under optimal conditions. Has been validated for use in flow cytometry. Each lot is quality control tested by immunofluorescent staining with flow cytometric analysis.

Eukaryotic cell lines

Policy information about cell lines

Cell line source(s)	ALMC-1 and ALMC-2 cell lines were shared by the laboratory of Diane F. Jelinek, Ph.D., from the Mayo Clinic. Diffuse large B cell lymphoma cell lines (SUDHL7 and OCI-Ly8) cell lines and multiple myeloma cell lines (AMO1, MM1S, HuNS, and RPMI-8226) were provided by Stephan Bohl from the laboratory of Anthony Letai at the Dana-Farber Cancer Institute.
Authentication	The AL cell lines were not authenticated as they were sent directly from the laboratory that produced them. Lymphoma and multiple myeloma cell lines were authenticated by short tandem repeat (STR) profiling.
Mycoplasma contamination	Cell lines were not tested for mycoplasma.
Commonly misidentified lines (See ICLAC register)	No commonly misidentified cell lines were used in this study.

Animals and other organisms

Policy information about studies involving animals; ARRIVE guidelines recommended for reporting animal research

Laboratory animals	NSG (NOD scid gamma) mice (Jackson Laboratory strain 005557) of both sexes, with ages ranging from 8-16 weeks, were used for ALMC-1 xenograft implantation by subcutaneous injections.
Wild animals	No wild animals were used in this study.
Field-collected samples	No field collected samples were used in this study.
Ethics oversight	All mouse work was completed under IACUC protocol #1095, approved by the Harvard Center for Comparative Medicine. Mice were housed with full access to water and chow and monitored daily after initial injection, taking daily tumor size and body weight measurements. Treatment began at 150 mm^3 and mice were euthanized before tumors reached 4000 mm^3.

Note that full information on the approval of the study protocol must also be provided in the manuscript.

Human research participants

Policy information about studies involving human research participants

Population characteristics	All patient information is available in supplemental table S1. Additional patient information is not available due to patient privacy concerns.
Recruitment	Eligible patients were identified based on their chart histories and the inclusion/exclusion criteria of the study. There was no outside outreach for recruitment and all patients will be recruited internally by study collaborators. We have no self-selection biases to declare - all consenting patients with established disease were accepted to the study.
Ethics oversight	Bone marrow aspirates were collected from patients with treatment-naïve and relapsed AL amyloidosis under protocol H-36533 at Boston Medical Center, which was approved by Panel Green Institutional Review Board (IRB) initially on 07/06/2017 and renewed annually for the duration of the studies.

Note that full information on the approval of the study protocol must also be provided in the manuscript.

Clinical data

Policy information about clinical studies

All manuscripts should comply with the ICMJE guidelines for publication of clinical research and a completed CONSORT checklist must be included with all submissions.

Clinical trial registration	*Provide the trial registration number from ClinicalTrials.gov or an equivalent agency.*
Study protocol	*Note where the full trial protocol can be accessed OR if not available, explain why.*
Data collection	*Describe the settings and locales of data collection, noting the time periods of recruitment and data collection.*
Outcomes	*Describe how you pre-defined primary and secondary outcome measures and how you assessed these measures.*

Dual use research of concern

Policy information about dual use research of concern

Hazards

Could the accidental, deliberate or reckless misuse of agents or technologies generated in the work, or the application of information presented in the manuscript, pose a threat to:

No	Yes	
☒	☐	Public health
☒	☐	National security
☒	☐	Crops and/or livestock
☒	☐	Ecosystems
☒	☐	Any other significant area

Experiments of concern

Does the work involve any of these experiments of concern:

No	Yes	
☒	☐	Demonstrate how to render a vaccine ineffective
☒	☐	Confer resistance to therapeutically useful antibiotics or antiviral agents
☒	☐	Enhance the virulence of a pathogen or render a nonpathogen virulent
☒	☐	Increase transmissibility of a pathogen
☒	☐	Alter the host range of a pathogen
☒	☐	Enable evasion of diagnostic/detection modalities
☒	☐	Enable the weaponization of a biological agent or toxin
☒	☐	Any other potentially harmful combination of experiments and agents

Could the accidental, deliberate or reckless misuse of agents or technologies generated in the work, or the application of information presented in the manuscript, pose a threat to:

No	Yes	
☒	☐	Public health
☒	☐	National security
☒	☐	Crops and/or livestock
☒	☐	Ecosystems
☒	☐	Any other significant area

Experiments of concern

Does the work involve any of these experiments of concern:

No	Yes	
☒	☐	Demonstrate how to render a vaccine ineffective
☒	☐	Confer resistance to therapeutically useful antibiotics or antiviral agents
☒	☐	Enhance the virulence of a pathogen or render a nonpathogen virulent
☒	☐	Increase transmissibility of a pathogen
☒	☐	Alter the host range of a pathogen
☒	☐	Enable evasion of diagnostic/detection modalities
☒	☐	Enable the weaponization of a biological agent or toxin
☒	☐	Any other potentially harmful combination of experiments and agents

Flow Cytometry

Plots

Confirm that:

☒ The axis labels state the marker and fluorochrome used (e.g. CD4-FITC).

☒ The axis scales are clearly visible. Include numbers along axes only for bottom left plot of group (a 'group' is an analysis of identical markers).

☒ All plots are contour plots with outliers or pseudocolor plots.

☒ A numerical value for number of cells or percentage (with statistics) is provided.

Methodology

Sample preparation	Bone marrow aspirates were collected from consenting patients at Boston Medical Center, yielding 2-5 mL in volume each. Samples were then transferred to the Harvard School of Public Health for processing and analysis. To isolate mononuclear cells, the samples were diluted in PBS to a total volume of 10 mL, then layed atop Ficoll Paque and centrifuged for 35 minutes at 300xg. Mononuclear cells were then isolated and a subset was taken to measure cell abundance and viability. 8x10^6 cells were immediately subjected to BH3 profiling. An additional 8x10^6 cells were then resuspended in AL media and plated in a 96-well plate prepared with drug treatments, with viability in response to drug treatments to be measured the following day.
Instrument	Attune NxT (Thermo Fisher, A24858)
Software	Attune NxT software (Thermo Fisher Scientific)
Cell population abundance	Typically, patient samples yielded 1-5% CD138+ cells. Samples with insufficient levels of CD138+ cells were excluded from the analysis.
Gating strategy	(See supplementary information for example)<br>Events above baseline FSC/SSC were labeled as cells, then discriminated by FSC-H/FSC-A to identify single cells. Plasma cells were then identified by positive CD138 (Pacific Blue) staining.<br>For BH3 profiling experiments, Cytochrome C loss or retention after peptide treatments was measured by Cytochrome C (Alexa Fluor 647) staining. Cells staining negatively for Cytochrome C have committed to apoptosis and are identified as such.<br>For ex vivo drug sensitivity assays, the level of induction of apoptosis in CD138+ plasma cells was measured by Annexin V (Alexa Fluor 647) positivity as a marker for apoptosis.

☒ Tick this box to confirm that a figure exemplifying the gating strategy is provided in the Supplementary Information.
